# Specificity and functional interplay between influenza virus PA-X and NS1 shutoff activity

**DOI:** 10.1371/journal.ppat.1007465

**Published:** 2018-11-29

**Authors:** Chutikarn Chaimayo, Megan Dunagan, Tsuyoshi Hayashi, Netty Santoso, Toru Takimoto

**Affiliations:** 1 Department of Microbiology and Immunology, University of Rochester Medical Center, Rochester, New York, United States of America; 2 Department of Microbiology, Faculty of Medicine Siriraj Hospital, Mahidol University, Bangkok, Thailand; University of Georgia, UNITED STATES

## Abstract

Influenza A viruses modulate host antiviral responses to promote viral growth and pathogenicity. Through viral PA-X and NS1 proteins, the virus is capable of suppressing host protein synthesis, termed “host shutoff.” Although both proteins are known to induce general shutoff, specificity of target genes and their functional interplay in mediating host shutoff are not fully elucidated. In this study, we generated four recombinant influenza A/California/04/2009 (pH1N1) viruses containing mutations affecting the expression of active PA-X and NS1. We analyzed viral growth, general shutoff activity, specificity of mRNA targets, and viral gene expressions. Our results showed that PA-X was the major contributor in reducing general host protein expression in the virus-infected cells. Intriguingly, our transcriptomic analysis from infected human airway A549 cells indicate that shutoff-active NS1 specifically targeted host mRNAs related to interferon (IFN) signaling pathways and cytokine release. Specificity of target mRNAs was less evident in PA-X, although it preferentially degraded genes associated with cellular protein metabolism and protein repair. Interestingly, in the presence of shutoff-active NS1, PA-X also degraded viral mRNAs, especially NS segments. The virus expressing shutoff-active NS1 with reduced amount of PA-X expression most efficiently suppressed antiviral and innate immune responses in human cells, indicating that influenza virus needs to optimize the contribution of these two shutoff proteins to circumvent host responses for its optimum growth.

## Author summary

Virus infection induces a wide range of host defense responses, such as the innate immune response and inflammation. Some viruses express accessory proteins to induce general shutoff of host protein synthesis, which is one of the major viral strategies to counteract host antiviral activity and immune response. Influenza A virus expresses two proteins, NS1 and PA-X, to induce general shutoff. Their mechanism of action is different; NS1 interacts with CPSF30 and blocks the 3’end processing of host pre-mRNAs, while PA-X degrades target mRNAs through its endonuclease domain. Importantly, the NS1 and PA-X activities differ among the virus isolates, suggesting a functional interplay between these two proteins. In this study, we rescued and characterized 2009 pandemic H1N1 mutant viruses with various NS1 and PA-X shutoff activities. Our data showed that PA-X was the major contributor of general shutoff of host protein synthesis, while NS1 specifically targeted genes involved in host innate response and cytokine response. Our data also suggest that viral mRNAs, especially the NS gene can be the target of PA-X induced degradation if NS1 shutoff activity is active. This study indicates a functional interplay between NS1 and PA-X proteins, which is required for optimum viral growth.

## Introduction

Influenza A virus is a major respiratory pathogen that frequently causes seasonal epidemics and periodic pandemics, resulting in half-a million deaths worldwide each year [1]. Like many other viruses, the influenza virus relies on the host cellular machinery to facilitate its replication cycle. However, host cells recognize and respond to viral infection through inducing various anti-viral proteins and interferons (IFNs). To overcome the host defense system, many viruses express proteins to evade the innate immune response or target general host protein synthesis. General shutoff targeting host mRNAs limits competition from cellular transcripts and secures priority access of viral mRNAs to host translation machinery [2].

Influenza A virus expresses two viral proteins, NS1 and PA-X, to induce host shutoff. The NS1 is a multifunctional protein best known for its role in antagonizing IFN responses by blocking RIG-I activation and preventing nuclear localization of transcription factors [3–5]. NS1 of human viruses also suppresses host protein synthesis by preventing maturation of host transcripts [6, 7]. Influenza NS1 has been shown to bind the 30-kDa subunit of CPSF (CPSF30), a component of CPSF complex involved in the cleavage of the 3' signaling region of newly synthesized pre-mRNA [6, 7]. A crystal structure of a complex composed of the NS1 effector domain (ED) and F2F3 fragment of CPSF30 indicates that two NS1 molecules and two CPSF30 are components of this complex, and NS1 residues F103, M106, K108, D125, and D189 play a critical role for CPSF30 interaction [8, 9]. These key NS1 residues are highly conserved among human isolates, although they are less conserved among animal isolates. For example, the pathogenic H5N1 influenza A/Hong Kong/483/97 (HK97) virus isolated from humans as well as a mouse-adapted A/Puerto Rico/8/34 (PR8) do not bind CPSF30 due to mutations at residues 103 and 106, and are not able to induce general shutoff [9, 10]. The NS1 of 2009 pandemic influenza viruses (pH1N1), including A/California/04/2009 (Cal) lack the ability to bind to CPSF30 due to mutations in the consensus CPSF30 binding residues (K108R, D125E and D189G), and therefore are unable to induce general shutoff [9].

PA-X is a novel influenza virus shutoff protein expressed from PA mRNA as a result of ribosomal frameshifting [11]. Although a limited amount of PA-X is expressed in infected cells due to low frameshift efficiency [11], PA-X significantly impacts general host shutoff and suppresses host antiviral and immune responses [11–16]. Importantly, PA-X shutoff activity is stronger than NS1 in the context of plasmid transfection [12]. PA-X contains the initial 191 amino acids of the PA protein and the unique C-terminal 41 or 61 amino acids, depending on the virus strains [17]. We and others demonstrated that the endonuclease active site, residing in the N-terminal domain of PA-X, is responsible for cellular mRNA degradation [11, 12, 14, 18, 19]. Our previous study showed that PA-X localized and degraded host mRNAs both in the nucleus and cytoplasm [12]. We and others also found that the unique C-terminal region is crucial for high shutoff activity [12, 20]. A recent study also suggests that PA-X selectively degrades RNA polymerase II-transcribed host mRNAs in the nucleus [19]. However, the exact molecular mechanism of PA-X mediated shutoff activity remains largely unknown. Furthermore, the expression of PA-X is highly conserved, but its shutoff activity varies between the strains. Human viruses seem to have reduced PA-X shutoff activity than avian viruses due to mutations in the N-terminal domain [18]. Importantly, the 2009 pH1N1 viruses express highly active PA-X, but their NS1 proteins lack shutoff activity [9, 12, 18]. Therefore, the variation of NS1 and PA-X activities between the viruses could promote the optimum viral growth in their specific hosts.

In this study, we investigated the effect of influenza PA-X and NS1 proteins on viral growth efficiency and suppression of host mRNAs in the context of virus infection. We generated recombinant influenza A viruses (A/California/04/2009) containing mutations in PA and NS genes. The viruses express shutoff-active or inactive forms of NS1 with a normal or reduced amount of PA-X expression. Using these viruses, we determined i) the impact of PA-X and NS1 shutoff activities on host protein synthesis, ii) the specificity of target host transcripts, iii) regulation of host innate immune responses, and iv) the effect of shutoff activities on viral mRNA expression. Our data indicated that i) PA-X suppressed host protein synthesis more efficiently than NS1, ii) shutoff-active NS1 specifically targeted genes involved in innate signaling and cytokine release, while target transcripts of PA-X were less specific, but showed some preference for genes involved in cellular protein metabolism and protein repair, and iii) in the presence of both active NS1 and PA-X, expression of viral transcripts, especially NS1 and NEP mRNAs, were substantially reduced. Our results suggest that influenza viruses regulate their PA-X and NS1 shutoff activities to create an optimum environment for efficient growth and spread in a specific host.

## Results

### Generation and characterization of recombinant viruses containing mutations in PA and/or NS genes

**Fig 1:**
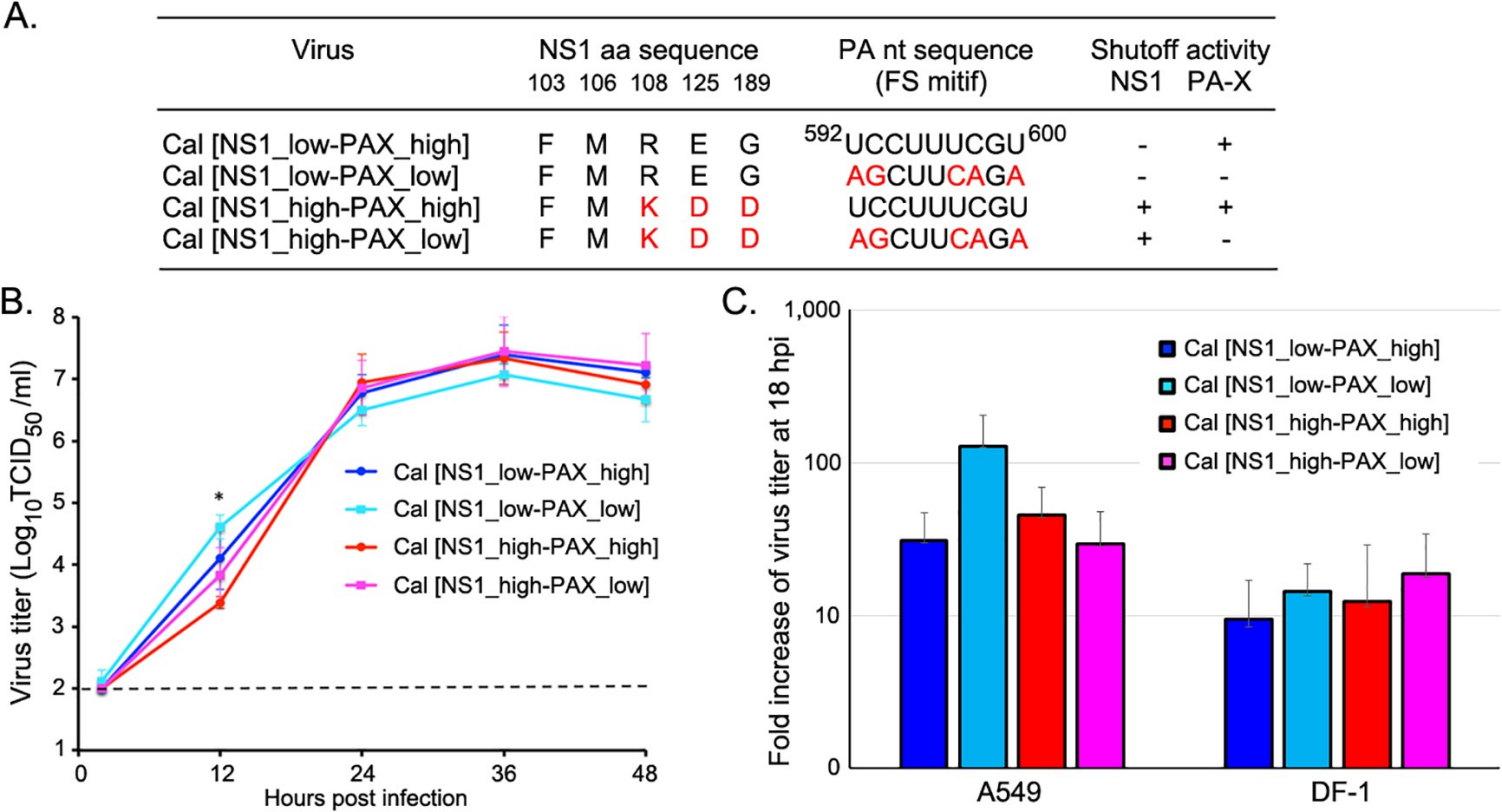
Generation of influenza PA-X and NS1 mutant viruses. (A) Cal mutant viruses contain mutations in either the frame-shift motif of PA gene or indicated NS1 amino acids or both. (B) Multi-step viral growth kinetics of the viruses in MDCK cells. Cells were infected with the viruses at an MOI of 0.05 and cultured in the presence of trypsin for up to 48 h. Virus samples in the cultures were titrated in MDCK cells. The dashed line indicates the detection limit of the assay. (C) A single-step virus growth in A549 and DF-1 cells. Cells were infected with the viruses at MOI 2 and cultured at 37°C. Released viruses in the culture supernatant were collected at 2 and 18 h pi and titrated. Fold increase of the virus titer at 18 h pi compared to 2 h pi was calculated. The data represent averages with standard deviations (n = 3). *, P< 0.05.

Both influenza NS1 and PA-X proteins are known contributors of host shutoff, although their mechanism of action is different. To analyze the functional interplay between PA-X and NS1, we generated viruses with various shutoff activities by mutating the PA-X and NS1 genes (Fig 1A). The pH1N1 strain we used (A/California/04/2009) expresses highly active PA-X, but shutoff-inactive NS1 (referred to as wild-type Cal [NS1_low-PAX_high]). To reduce expression of PA-X, we inserted mutations in the frameshift motif of Cal PA (referred to as Cal [NS1_low-PAX_low]), which we previously confirmed to significantly reduce PA-X expression in infected cells [14]. To activate NS1 shutoff activity, we made three mutations in the Cal NS1 gene (R108K, E125D, G189D), which were shown to enhance binding to CPSF30 [9] and suppress the expression of co-expressed proteins [9, 12]. Two mutant viruses, Cal [NS1_high-PAX_high] (expresses shutoff-active NS1) and Cal [NS1_high-PAX_low] (expresses shutoff-active NS1 with limited amount of PA-X) were successfully rescued. These viruses were plaque-cloned in MDCK cells, and their PA and NS gene sequences were confirmed to have the designed mutations. Together with the Cal wild-type (Cal [NS1_low-PAX_high]) and Cal [NS1_low-PAX_low] (reduced PA-X expression) we previously rescued [14], we analyzed the effect of the viral shutoff activities on host response and virus growth.

**Fig 2:**
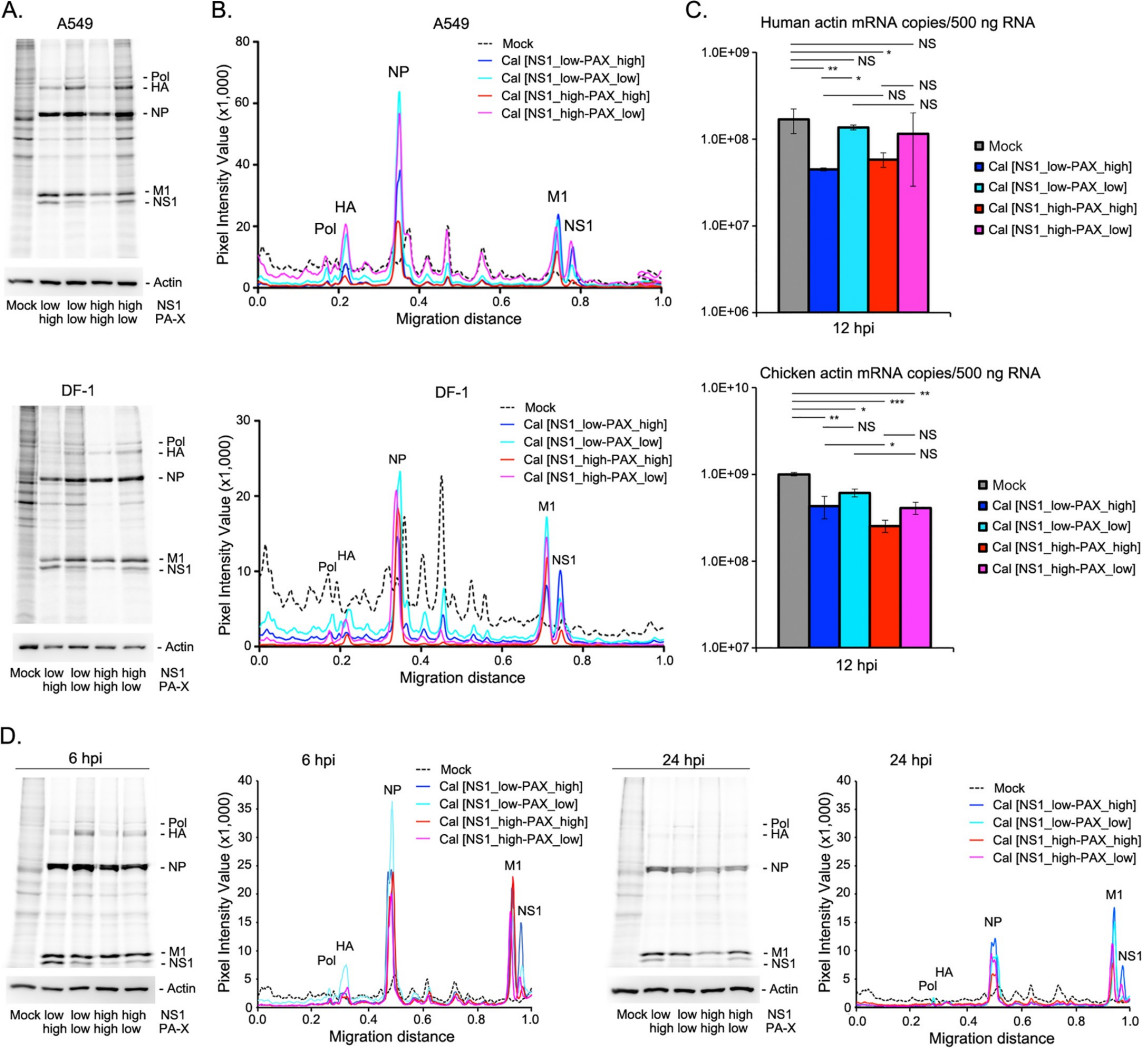
Shutoff activity on host protein synthesis of human and avian cultured cells by mutant viruses. (A) A549 or DF-1 cells were left uninfected or infected with the viruses at MOI of 2. At 16 h pi, cells were labeled with ^35^S-Met/Cys for 30 min and the radiolabeled lysates were resolved by SDS-PAGE. As loading controls, cellular actin was detected by Western blot analysis (lower panel). (B) Densitometric traces of labeled proteins shown in (A). (C) Quantities of cellular β-actin mRNAs in infected A549 or DF-1 cells at 12 h pi as determined by qRT-PCR. The data represent averages with standard deviations (n = 3). *, P< 0.05, **, P<0.01, ***, P<0.001, NS, not significant. (D) Shutoff activity of the viruses in A549 cells at an early and late time points. Cells were labeled with ^35^S-Met/Cys at 6 or 24 h pi and total lysates were analyzed by SDS-PAGE as (A).

First, we determined multi-step growth kinetics of the viruses at various time points (2, 12, 24, 36, and 48 h post infection (pi)) in Madin-Darby Canine Kidney (MDCK) cells. While Cal [NS1_low-PAX_low] virus grew better than other viruses at 12 h pi, all recombinant viruses reached the plateau phase at approximately the same time point after infection with the equivalent virus titer (Fig 1B). We also determined the production of infectious virions from infected human lung epithelial A549 and chicken fibroblast DF-1 cells. Similar to what was observed in MDCK cells, infected A549 cells yielded more virion production of Cal [NS1_low-PAX_low] than other viruses although there was no statistically significant difference (Fig 1C). Next, we determined the effect of the mutations on cellular protein synthesis in infected A549 cells and DF-1 cells. The host proteins were metabolically labeled with ^35^S-Met/Cys for 30 min at 16 h pi. Viruses expressing normal level of PA-X (Cal [NS1_low-PAX_high], Cal [NS1_high-PAX_high]) efficiently suppressed host protein synthesis in A549 cells (Fig 2A and 2B). Levels of newly synthesized host proteins detected by metabolic labeling also correlated with cellular β-actin mRNA levels as determined by qRT-PCR (Fig 2C), which demonstrated less actin mRNA levels in Cal [NS1_low-PAX_high] or Cal [NS1_high-PAX_high] infected A549 cells. Interestingly, viruses expressing mutant NS1 induced stronger general host shutoff in DF-1, but not in A549 cells (Cal [NS1_high-PAX_high] compared to Cal [NS1_low-PAX_high] or Cal [NS1_high-PAX_low] compared to Cal [NS1_low-PAX_low]). These results indicate that PA-X has stronger impact than NS1 in inducing general protein expression in human A549 cells, while NS1 also contributes to general shutoff in avian DF-1 cells.

We also determined the shutoff activities at various time points to assess the dynamics of the effect in protein expression in A549 cells. The shutoff activity was detected as early as 6 h pi, especially in cells infected with the viruses that express either shutoff-active NS1 or PA-X, or both (Fig 2D). At 24 h pi, the difference of host shutoff activity between the viruses was less evident, likely reflecting the additional effect of virus infection in host protein synthesis, such as RNA pol II degradation [21, 22].

### Transcriptomic profiling of A549 cells infected with influenza NS1 and PA-X mutant viruses

Although both influenza NS1 and PA-X proteins confer general suppression of host protein synthesis, they employ dissimilar mechanisms to reduce host gene expression, suggesting that their target mRNAs could be different [7, 11, 12, 19, 20]. Therefore, we determine the preferential cellular mRNA targets of NS1 and PA-X by comparing the mRNA expression profile of human A549 cells left uninfected (mock) or infected with the various recombinant viruses for 16 h. First, we validated the RNA samples used for the RNA-Seq experiment. Processing statistics for transcriptomic analysis show mapped sequence reads of each sample ranging from 77–99% with the uniquely mapped sequence reads ranging from 67–90% (S1A Fig). Sample-to-sample distance matrix showed a high degree of similarity between the three replicates of each condition and some degree of similarity among all virus-infected conditions, but was highly distinct from mock samples (S1B Fig). Principal component analysis was performed to visualize the overall effect of experimental covariates and batch effects. The sample groups were separated following the PC1 and PC2 axes, indicating different gene expression profiles between mock and infected conditions and also between the four different virus-infected conditions. Among the four viruses, Cal [NS1_high-PAX_high] and Cal [NS1_high-PAX_low] gene expression profiles were the most closely related (S1C Fig).

In our RNA-Seq analysis, 34,677 annotated genes showed non-zero total read count. Volcano plots were created to graphically display the significance and magnitude of changes in the data points between each pair of viruses to mock. The majority of differentially expressed genes were up-regulated upon infection (red dots), while down-regulated genes (green dots) were present to the lesser extent in all virus-infected conditions compared to mock (S2A Fig). Differential gene expression analysis of these 34,677 genes indicated that the number of genes significantly up-regulated (adjusted P-value < 0.05) at least two-fold in 16 h pi-virus infected cells compared to mock were 1,450 (4.18%), 1,218 (3.51%), 945 (2.73%), and 780 (2.25%) for Cal [NS1_low-PAX_high], Cal [NS1_low-PAX_low], Cal [NS1_high-PAX_high], and Cal [NS1_high-PAX_low], respectively (S2B Fig). Among these, 503 genes were commonly up-regulated upon infection with the viruses, while other genes were specifically up-regulated in certain virus infected conditions (S2C Fig left). Similarly, a number of genes were significantly down-regulated (adjusted P-value < 0.05) at least two-fold in virus infected cells compared to mock: 465 (1.34%), 372 (1.07%), 224 (0.65%), and 142 (0.41%) for Cal [NS1_low-PAX_high], Cal [NS1_low-PAX_low], Cal [NS1_high-PAX_high], and Cal [NS1_high-PAX_low], respectively (S2B Fig). Among these, 53 genes were commonly down-regulated upon infection with any recombinant viruses (S2C Fig right).

Next, we directly compared differentially expressed genes (DEGs) between cells infected with recombinant viruses to pinpoint the outcome of PA-X and active NS1 on host gene expression (Table 1). PA-X reduced the expression of 55 (Cal [NS1_low-PAX_high] vs. Cal [NS1_low-PAX_low]) and 127 (Cal [NS1_high-PAX_high] vs. Cal [NS1_high-PAX_low]) genes at least two-fold, but also increased the expression of 211 (Cal [NS1_low-PAX_high] vs. Cal [NS1_low-PAX_low]) and 137 (Cal [NS1_high-PAX_high] vs. Cal [NS1_high-PAX_low]) genes, suggesting that PA-X also increases expression of certain host genes. Interestingly, while expression of mutant NS1 that can induce general shutoff insignificantly, increased the numbers of at least two-fold-upregulated genes (eight DEGs for Cal [NS1_high-PAX_high] vs. Cal [NS1_low-PAX_high] and 17 DEGs for Cal [NS1_high-PAX_low] vs. Cal [NS1_low-PAX_low]), it markedly increased the numbers of at least two-fold-downregulated genes in infected cells regardless of PA-X expression (288 DEGs for Cal [NS1_high-PAX_high] vs. Cal [NS1_low-PAX_high] and 282 DEGs for Cal [NS1_high-PAX_low] vs. Cal [NS1_low-PAX_low]) (Table 1). These results suggest that the target genes of PA-X, but not shutoff-active NS1 increase the expression of some other host transcripts.

**Table 1:** Number of differentially expressed genes between virus-infected cells.

Comparison	Number of DEGs^1)^	Number of DEG (> 2-fold) Up Down
Cal [NS1_low-PAX_high] vs. Cal [NS1_low-PAX_low]	4,847	211 55
Cal [NS1_high-PAX_high] vs. Cal [NS1_high-PAX_low]	7,420	137 127
Cal [NS1_high-PAX_high] vs. Cal [NS1_low-PAX_high]	3,245	8 288
Cal [NS1_high-PAX_low] vs. Cal [NS1_low-PAX_low]	5,853	17 282
Cal [NS1_high-PAX_high] vs. Cal [NS1_low-PAX_low]	4,052	8 167
Cal [NS1_high-PAX_low] vs. Cal [NS1_low-PAX_high]	8,503	364 551

^1)^Adjusted *p*-value <0.05 (from 34,677 genes)

### Shutoff-active NS1 specifically targets genes involved in IFN sensing and signaling, inflammatory response, and chemokine/cytokine-mediated signaling pathways

**Fig 3:**
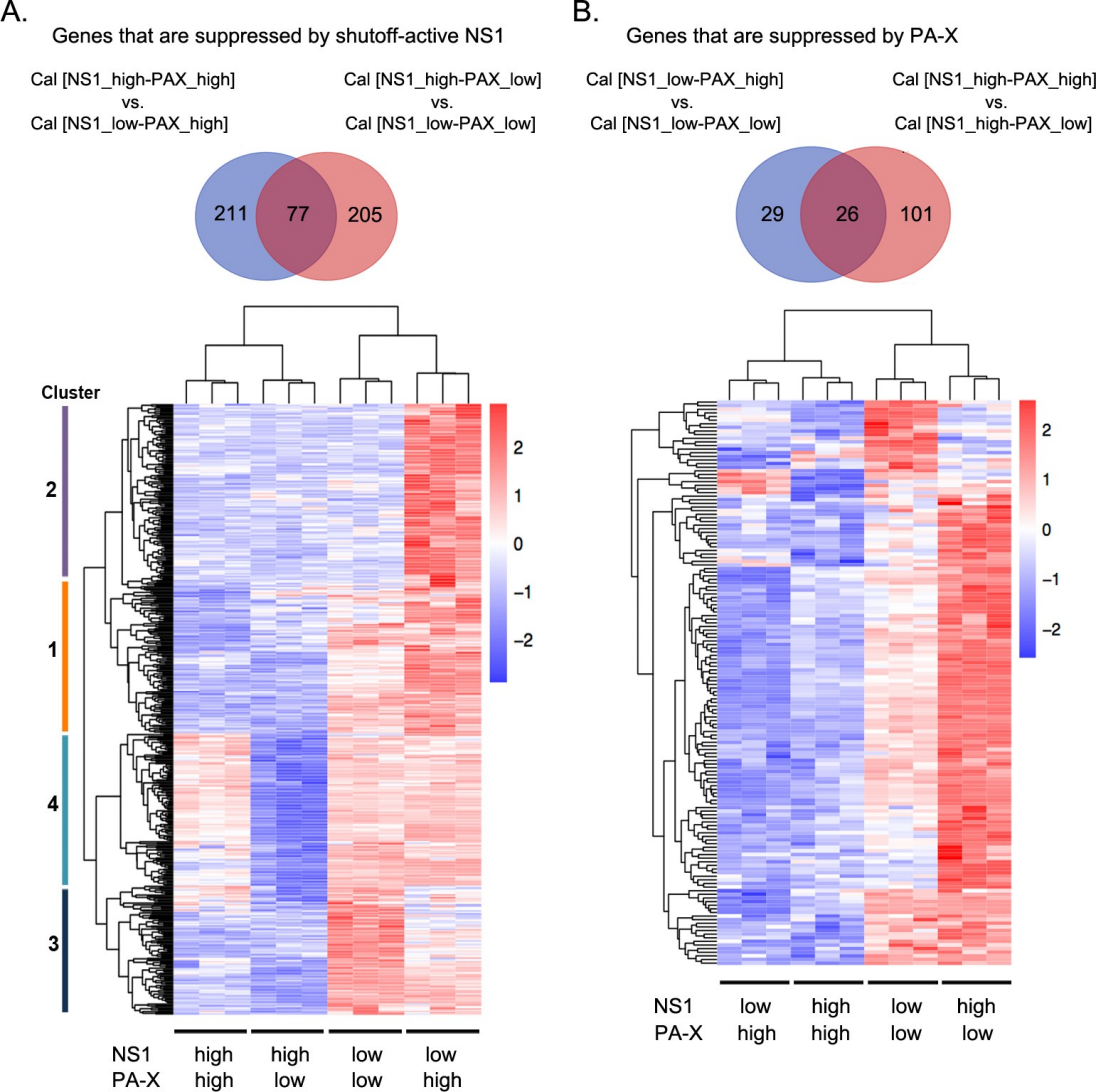
Analysis of genes suppressed by active NS1 or PA-X. Genes suppressed by shutoff-active NS1 (A) or PA-X (B) are shown. A Venn diagram demonstrates number of genes suppressed by NS1 or PA-X at least two-fold. Heatmaps show the 493 (A) or 156 (B) DEGs. Each row represents each gene (blue: low expression; red: high expression; Z-score scaling from -2 to 2). Each column represents one replicate from each virus group. Shutoff activity elicited from PA-X and active NS1 was noted under each virus-infected conditions. Hierarchical clustering was performed and cluster numbers based on their expression profiles were demonstrated on the left of the heatmap (A).

**Fig 4:**
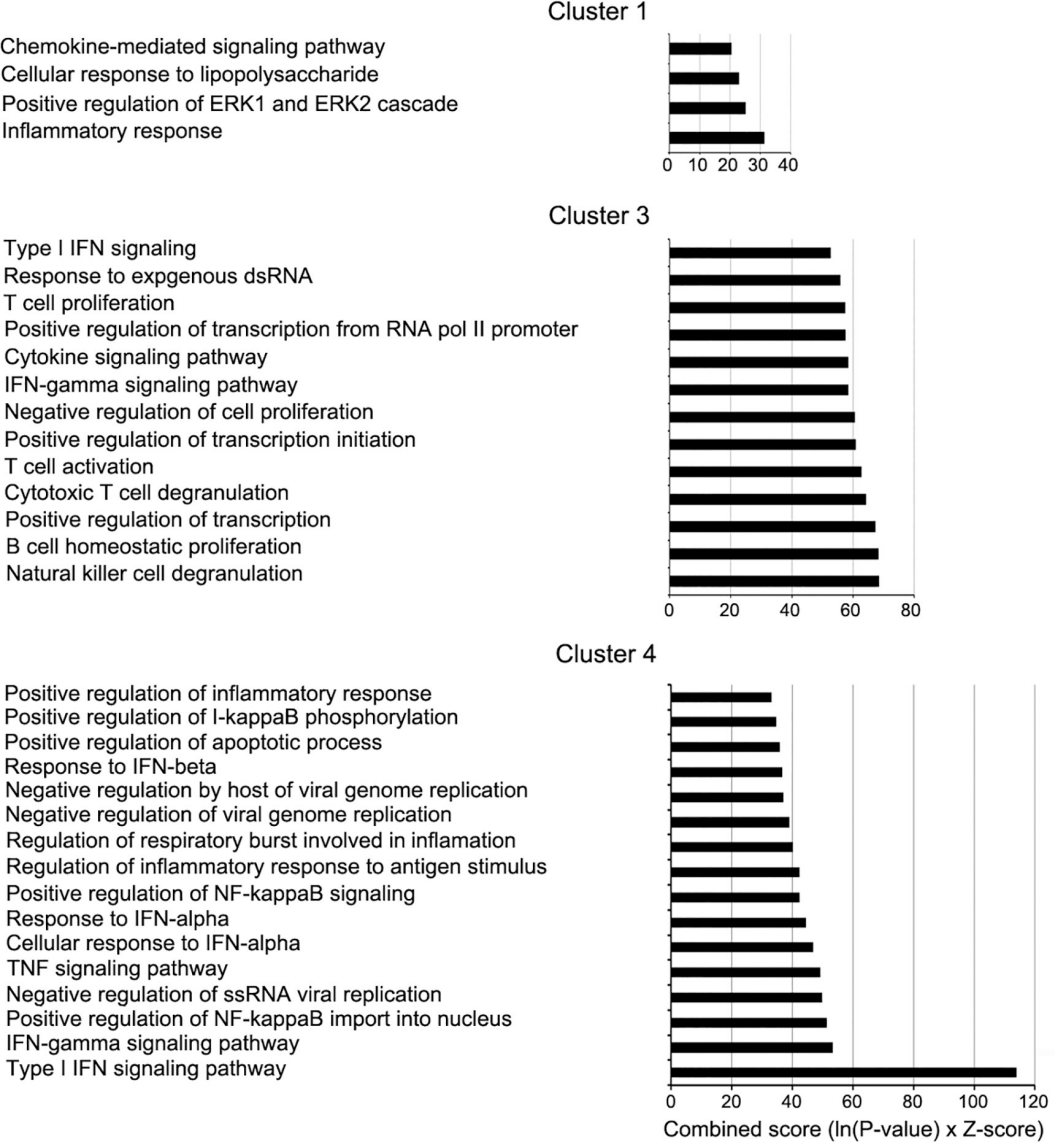
Clustering and pathway analysis of genes suppressed by active NS1. Bar graphs demonstrate the enriched biological processes associated with cluster 1, 3, and 4 in Fig 3, which were plotted against the combined score (Enrichr) on the y-axis. Cluster 2 showed no significant enriched biological process.

Next, we compared the differentially expressed genes between each pair of viruses whose difference was only in their NS1 shutoff activity. We identified 288 genes by comparing Cal [NS1_high-PAX_high] to Cal [NS1_low-PAX_high], and 282 genes by comparing Cal [NS1_high-PAX_low] to Cal [NS1_low-PAX_low]. There were 77 common genes between these two gene sets, and a total of 493 genes specifically downregulated by shutoff-active NS1, majority of which are genes induced by viral infection (Table 1, S1 Table). Strikingly, Gene ontology (GO) term analysis using Enrichr GO Biological Process 2017b database of these 493 genes indicated that the type I IFN signaling pathway, response to exogenous dsRNA, inflammatory response, and chemokine/cytokine-mediated signaling pathways were the primary biological pathways affected by infection with viruses possessing shutoff-active NS1 (S2 Table). We then compared the expression profiles of these 493 genes by constructing a heat map. Hierarchical clustering was performed to identify four different groups in which the differentially expressed genes belong to (Fig 3A and S1 Table). GO term analysis for genes in each cluster indicated difference in their specificity. Cluster 1 (contains 115 genes) were enriched with genes associated with the inflammatory response and the chemokine-mediated signaling pathway (Fig 4). Cluster 2 contained 151 genes that were expressed less in cells infected with all the mutant viruses compared to wild-type Cal [NS1_low-PAX_high] virus. However, genes in this cluster showed no significant association with any particular pathway (S1 Table). Cluster 3 contained 104 genes that were expressed less in Cal [NS1_high-PAX_high] and Cal [NS1_high-PAX_low] infected cells than those in Cal [NS1_low-PAX_low] and Cal [NS1_low-PAX_high] infected cells. These genes included those associated with response to exogenous dsRNA, type I IFN signaling pathway and positive regulation of transcription (Fig 4). Lastly, cluster 4 contained 123 genes whose expression levels were uniquely reduced by infection with Cal [NS1_high-PAX_low] relative to infection with Cal [NS1_high-PAX_high], Cal [NS1_low-PAX_low] and Cal [NS1_low-PAX_high] viruses. These genes were associated with the type I IFN signaling pathway, antiviral genes, tumor necrosis factor (TNF) signaling pathway, regulation of NF-κB signaling, apoptotic process, and inflammatory response (Fig 4). Collectively, this data suggest that three mutations in NS1 (R108K, E125D, G189D) which activate shutoff activity through CPSF30 binding also increased the virus’s ability to specifically suppress induction of genes involved in signaling pathways of the host innate immune response. The data of cluster 4, which showed reduced expression of host genes after infection with Cal [NS1_high-PAX_low] compared to Cal [NS1_high-PAX_high], also suggest that PA-X may interfere with NS1’s activity to specifically suppress genes involved in type I IFN signaling pathway (Fig 3A).

### PA-X preferentially suppressed cellular mRNAs related to cellular protein modification process, cellular protein metabolism and protein repair

To identify the mRNAs preferentially targeted by PA-X, we directly compared the downregulated genes between each pair of viruses whose difference was only in their PA-X shutoff activity. We identified 55 and 127 transcripts, which are uniquely suppressed at least two-fold by PA-X in the absence or presence of shutoff-active NS1, respectively (Fig 3B and Table 1). Twenty-six genes were common between the two gene sets (Fig 3B). We compared the expression profiles of these genes in infected cells by constructing a heat map (Fig 3B and S3 Table). GO term analysis of these 156 genes demonstrated enrichment in cellular protein modification process, cellular protein metabolism, and protein repair, suggesting that PA-X preferentially targets mRNA related to these pathways (S4 Table). The preferential targeting of mRNAs involved in cellular protein metabolism revealed by the GO term analysis may suggest an additional strategy of viruses possessing high PA-X activity to induce stronger inhibition of host protein synthesis (Fig 2).

### Impact of PA-X and NS1 on suppressing expression of genes involved in innate and cytokine responses

**Fig 5:**
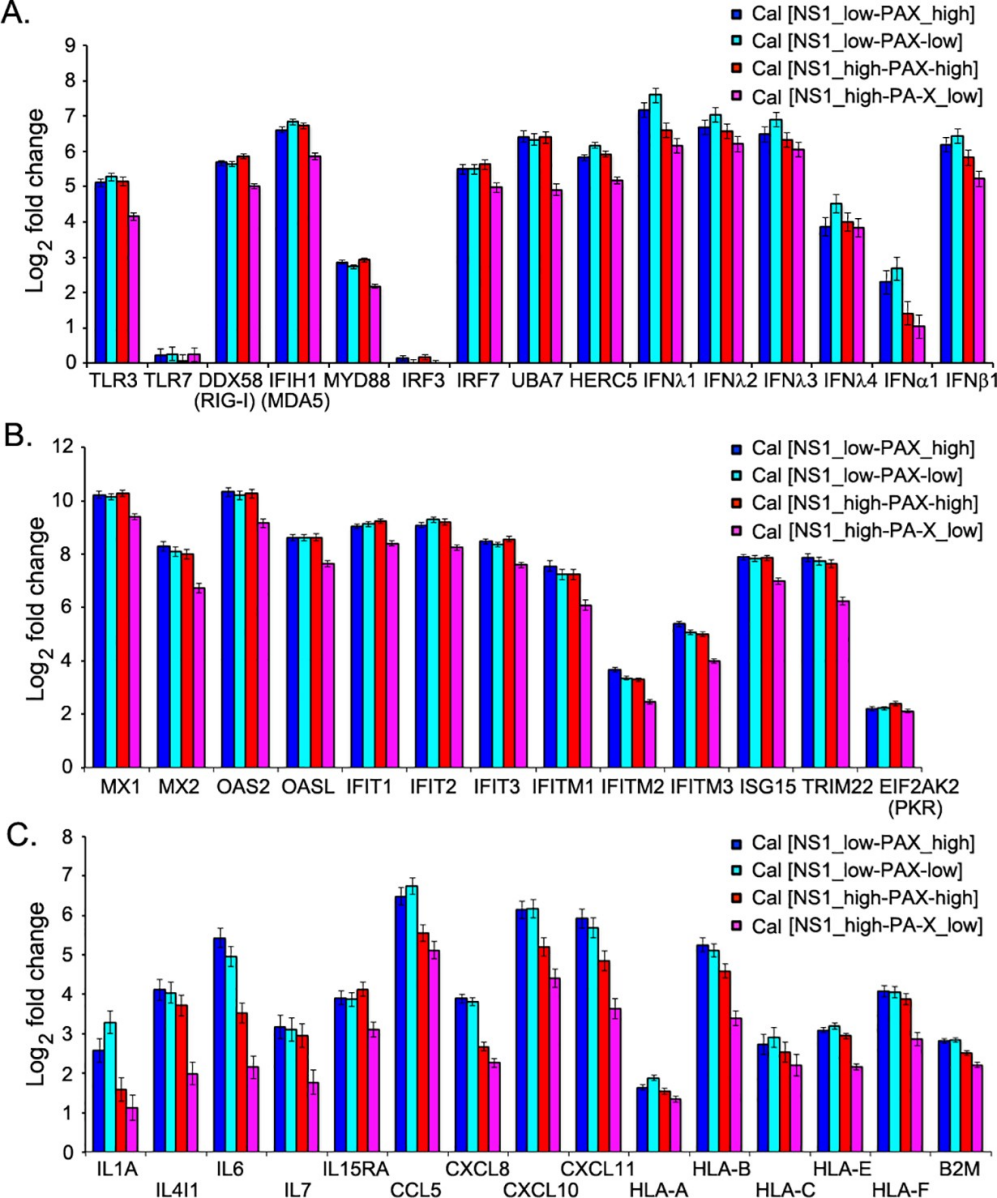
Induction of representative genes in infected cells. Fold change of genes involved in (A) innate signaling and IFNs, (B) ISGs, and (C) cytokines, chemokines and MHC class I molecules are shown. Bar graphs demonstrate fold change of the host genes in response to the virus infection compared to mock as revealed by the RNA-Seq analysis.

We next compared the induction of key genes known to be involved in innate response upon influenza virus infection. We selected the representative genes of innate signaling, IFN and IFN-stimulated genes (ISGs) and determined their induction upon infection. The expression of RIG-I signaling or antiviral response-related genes, such as DDX58 (RIG-I dsRNA sensing), IFIH1 (MDA-5 dsRNA sensing), IRF-7 (IFN regulatory factor 7; activates both IFN-α and IFN-β), UBA7 (ubiquitin activating enzyme that catalyzes ISGylation of targeted proteins), and HERC5 (ubiquitin protein ligase that mediates ISGylation of targeted proteins) were significantly elevated after infection by all recombinant influenza Cal viruses (Fig 5A). Similarly, a strong induction of type I (β) and type III (λ) IFNs was observed in infected as expected from a previous study [23]. Comparison between Cal [NS1_low-PAX_high] and Cal [NS1_low-PAX_low] suggest that PA-X contributes to the suppression of IFN-β and IFN-λ induction. However, induction of IFN genes or genes associated with the innate signaling pathway was most effectively suppressed by Cal [NS1_high-PAX_low], which has shutoff-active NS1 but reduced PA-X expression (Fig 5A). Influenza infection also induces ISGs, some of which were reported to be directly induced by viral infection despite the absence of IFN production [24]. Many ISGs possess antiviral effector function, which in turn limits the efficiency of virus growth and spread [25, 26]. We determined the induction of the ISGs upon infection with the viruses. Among the selected ISGs, Mx1, Mx2, OAS2, OASL, IFIT1, IFIT2, IFIT3, IFITM1, TRIM22, and ISG15 were highly up-regulated (130 to 1,300-fold change upon infection with wild-type Cal [NS1_low-PAX_high] virus), most of which are known to inhibit influenza virus infection and/or replication (Fig 5B). PA-X barely impacted the gene expression of the ISGs that were strongly induced upon influenza virus infection (Fig 5B and S3 Table). Little difference in gene level was observed between Cal [NS1_low-PAX_high] and Cal [NS1_low-PAX_low] virus-infected samples. Remarkably, Cal [NS1_high-PAX_low], again most effectively suppressed the ISG induction in infected cells, while Cal [NS1_high-PAX_high], which express both shutoff-active NS1 and PA-X, was less efficient, indicating the negative impact of PA-X on NS1-induced suppression of ISG induction (Fig 5B).

In response to viral infection, lung epithelial cells and leukocytes produce chemokines and other pro-inflammatory cytokines, which function in cell signaling to limit infection [27]. It is well established that regulation of cytokine and chemokine production plays a major role in viral pathogenicity and immune response [28]. In addition, antigen presentation by MHC class I proteins contribute to the clearance of infected cells by cytotoxic T cells [29, 30]. Therefore, we compared the differential expression of representative interleukin, chemokine, and MHC class I genes, which were significantly up-regulated upon infection. Among the selected interleukin genes, IL-6, which functions in acute inflammation and B-cell maturation, was highly up-regulated (40-fold change upon infection with wild-type Cal [NS1_low-PAX_high] virus). However, the Cal [NS1_high-PAX_low] virus induced only a four-fold change of IL-6 mRNA (Fig 5C). Among the selected chemokine genes, CCL5 (RANTES; chemoattractant for monocytes, memory T-helper cells, and eosinophils), CXCL10 (IP-10; chemoattractant for monocytes and T cells), and CXCL11 (IP-9; chemoattractant for interleukin-activated T cells) were highly up-regulated (60 to 90-fold change upon infection with wild-type Cal [NS1_low-PAX_high] virus) (Fig 5C). Importantly, viruses possessing shutoff-active NS1, especially Cal [NS1_high-PAX_low] virus, strongly inhibited the induction of interleukins and chemokines (Fig 5C).

Apart from host components involved in inflammatory and IFN-signaling pathways, MHC molecules are the crucial components of the immune system, as they link the innate and adaptive immune systems [29]. In response to virus infection, MHC class I molecules are responsible for presenting peptides derived from intracellular viral antigens to cytotoxic T cells. The MHC or human leukocyte antigen (HLA) class I heterodimer is assembled from a polymorphic heavy chain (HLA-A, HLA-B, HLA-C, HLA-E, HLA-F, and HLA-G) and a light chain called β2-microglobulin (β2m) [30]. Among the selected MHC class I genes, HLA-B was highly up-regulated (40-fold change upon infection with wild-type Cal [NS1_low-PAX_high] virus) and its expression level was lower in Cal [NS1_high-PAX_low] virus compared to other virus-infected conditions (Fig 5C).

### Verification of host gene expression by qRT-PCR and ELISA

**Fig 6:**
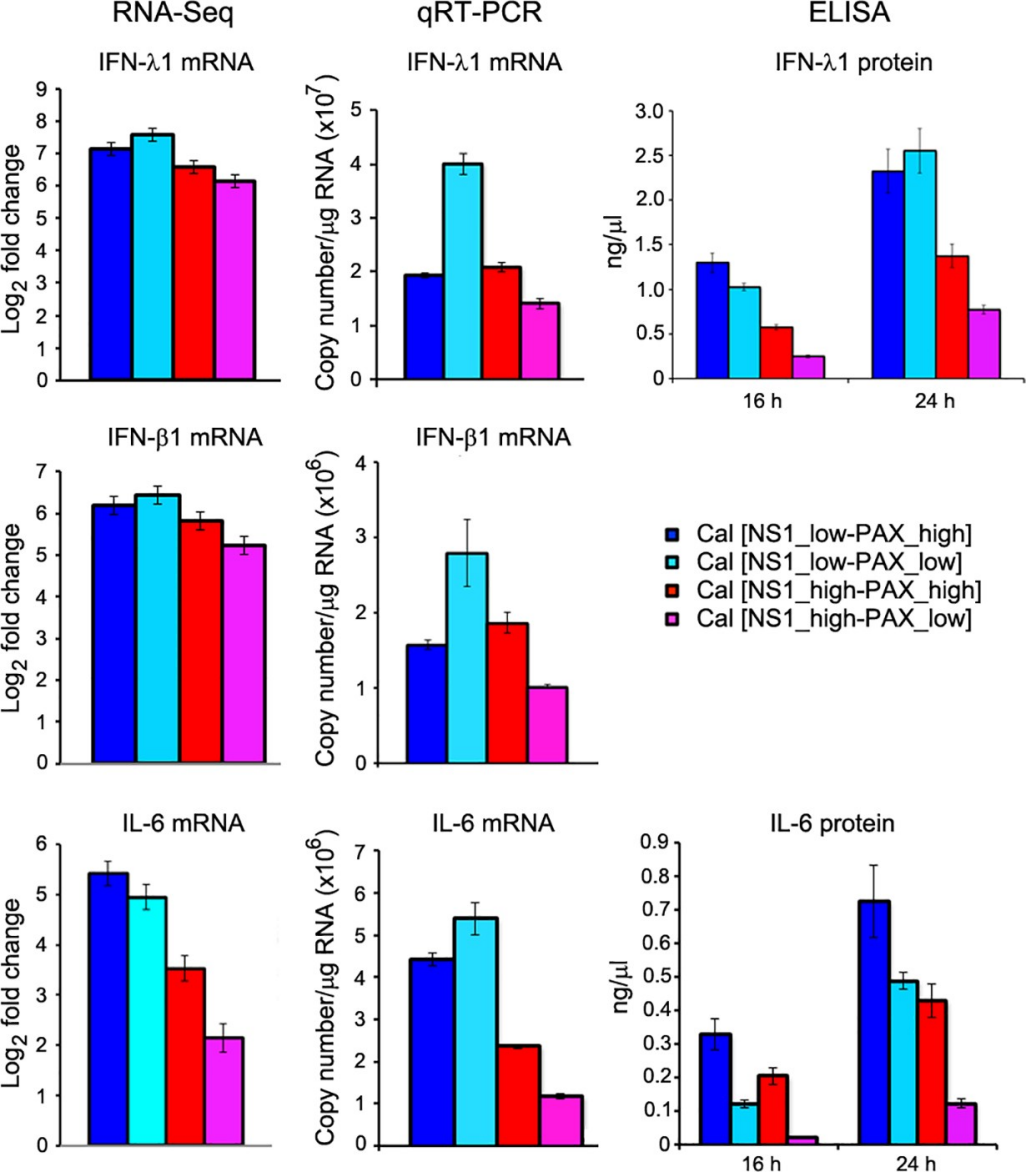
Validation of RNA-Seq data by qRT-PCR and ELISA. IFN-λ1, IFN-β1, and IL-6 mRNAs were quantitated by qRT-PCR and compared with RNA-Seq data. Secreted IFN-λ1 and IL-6 in the culture medium were quantified by ELISA at 16 and 24 h pi.

We next verified the expression levels of IFN-λ1, IFN-β1, and IL-6 mRNAs in infected cells by qRT-PCR. Total RNAs extracted from infected A549 cells were prepared at 16 h pi, and quantities of IFN-λ1, IFN-β1 and IL-6 mRNAs were measured. We observed a similar trend with RNA-Seq data that the Cal [NS1_high-PAX_low] virus most effectively suppressed the expression of the IFN-λ1, IFN-β1 and IL-6 mRNAs (Fig 6). We also measured the secreted IFN-λ1 and IL-6 proteins in culture supernatants of infected cells by sandwich ELISA. Again, the level of the secreted IFN-λ1 and IL-6, especially at 24 h pi, correlated well with our RNA-Seq result (Fig 6). Our finding indicates a significant role of shutoff-active NS1 in suppression of key host genes involved in innate and adaptive immune responses. However, this effect of shutoff-active NS1 was lessened by PA-X. Hence, it is conceivable that a functional interplay between PA-X and NS1 is required for the optimum regulation of host antiviral responses.

### Anti-innate activity of NS1 mutant

**Fig 7:**
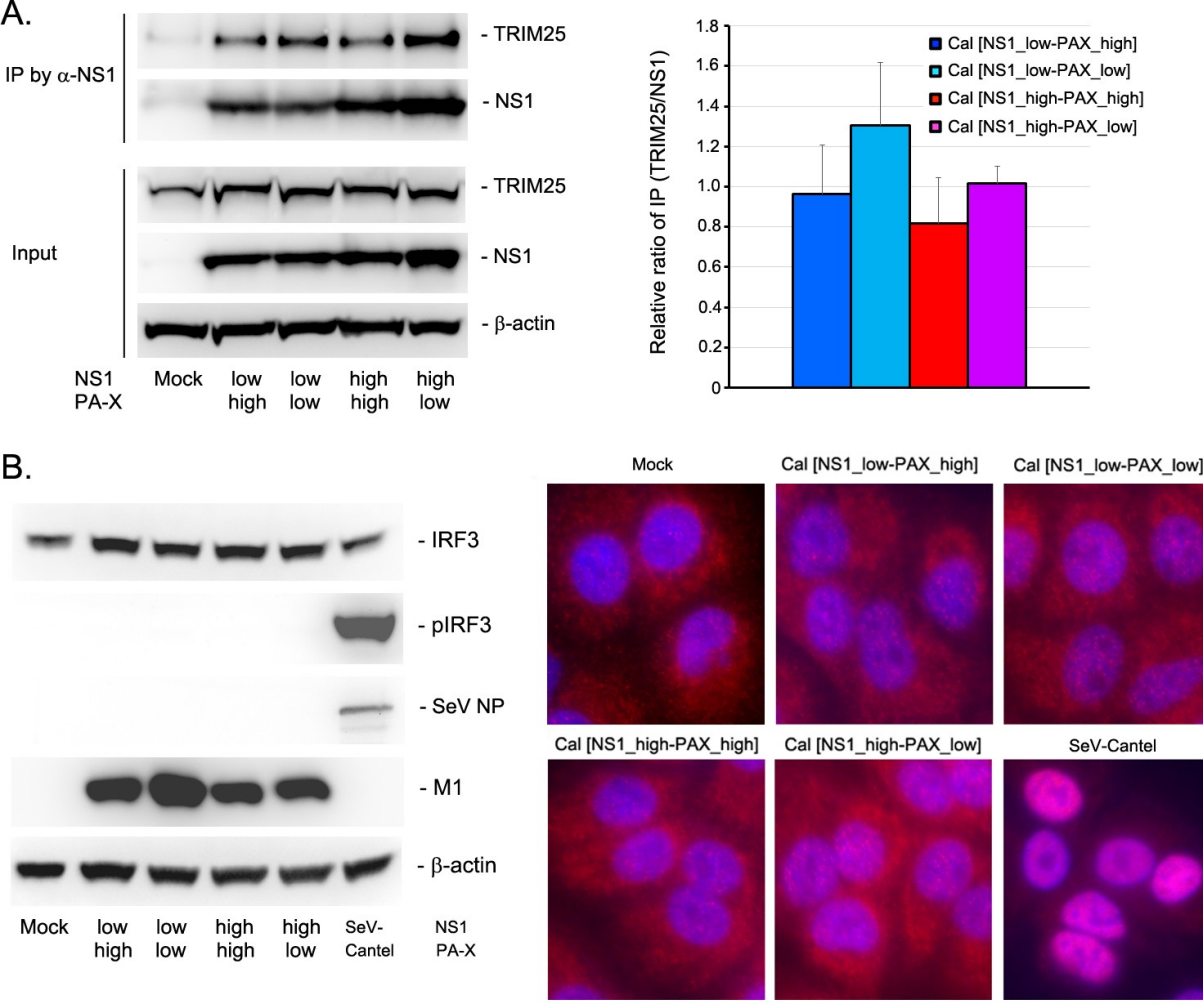
Inhibitory activity of NS1 on innate signaling. (A) Interaction between NS1 and TRIM25 was determined by co-immunoprecipitation assay. Infected cell lysates were reacted with anti-NS1 antibody, and the immunoprecipitated materials were analyzed for the presence of TRIM25 and NS1. Left figure is a representative of four independent experiments and bar graph on right shows relative ratio of co-immunoprecipitated proteins (n = 4). (B) Activation of IRF3 in infected cells. (Left) A549 cells infected with the viruses were determined for IRF phosphorylation by Western blot analysis. (Right) Translocation of IRF3 to the nucleus in infected cells was determined by immunofluorescence assay using anti-IRF3 antibody (red) counterstained with DAPI (blue).

The RNA-Seq and qRT-PCR data clearly indicate that the shutoff-active NS1 more specifically and effectively targets and suppresses innate immune responses than shutoff-inactive NS1. The specificity of NS1’s target mRNAs cannot be entirely explained by the activity of NS1 in inhibiting general pre-mRNA processing through its CPSF30 interaction. It is also possible that the three mutations introduced to NS gene (R108K, E125D, G189D) affect other functions of NS1 beyond its ability to interact with CPSF30. NS1 is well characterized for its inhibitory activity on innate signaling through its interaction with TRIM25, which ubiquitinate the RIG-I CARD domain [31, 32]. To determine if NS1 binding to CPSF30 affect the innate signaling pathway, we first analyzed the interaction between NS1 and TRIM25 by co-immunoprecipitation assay from the lysates of infected cells. Our data indicate that TRIM25 was co-immunoprecipitated with NS1 regardless of the mutation (Fig 7A). We did not detect any significant difference in the amount of TRIM25 co-immunoprecipitated with NS1 wild-type and the mutant. We next determined activation and phosphorylation of IRF-3 at early time points after infection. In contrast to Sendai virus Cantel strain that is known to be a strong inducer of innate response [33], both wild-type and mutant NS1 efficiently blocked the IRF3 phosphorylation and translocation to the nucleus (Fig 7B), which is consistent with the previous report [34]. These results indicate that both wild-type and mutant NS1 are capable of blocking IRF3 activation in infected cells. Therefore, the specific suppression of innate gene expression through NS1 mutation detected in transcriptomic analysis likely reflect downstream effect of NS1-CPSF30 complex possibly during transcription of the innate genes.

### PA-X suppressed NS mRNA expression in the presence of shutoff-active NS1

**Fig 8:**
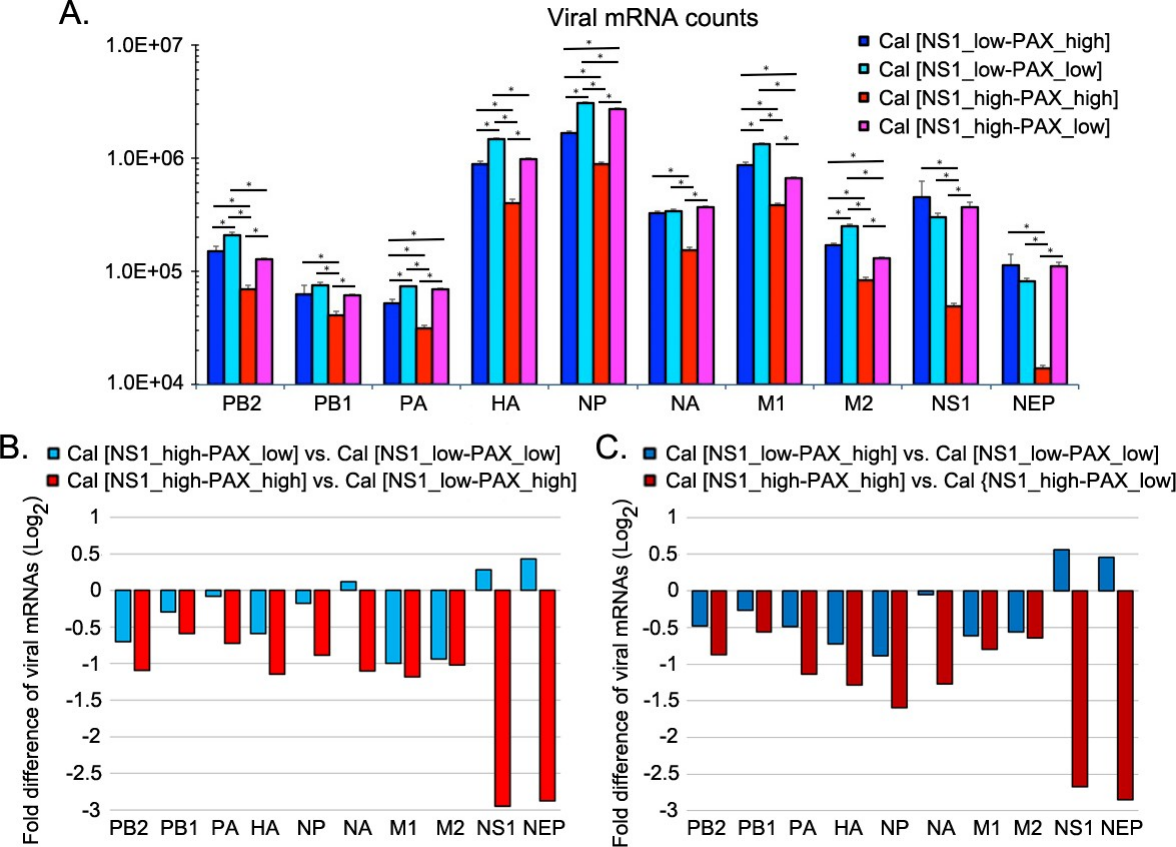
RNA-Seq analysis of influenza viral gene expression. (A) Viral gene expression data obtained from RNA-Seq analysis demonstrates levels of normalized influenza viral mRNA counts of PB2, PB1, PA, HA, NP, NA, M1, M2, NS1, and NEP mRNAs. DESeq2 performs an internal normalization where geometric mean is calculated for each gene across all infected samples. (B) Impact of shutoff-active NS1 on viral gene expression was analyzed by a direct comparison between viruses expressing shutoff-active and inactive NS1, and was demonstrated in fold difference of viral mRNAs in log_2_ scale. (C) Impact of PA-X on viral gene expression was analyzed by a direct comparison between viruses expressing normal and reduced amount of PA-X, and was demonstrated in fold difference of viral mRNAs in log_2_ scale.

The transcriptome data suggest that Cal NS1 mutations at the CPSF30 binding residues (R108K, E125D, G189D) enhance specific suppression of host innate and cytokine responses. However, PA-X negatively affected NS1’s activity in regulating host responses (Figs 5 and 6). We speculated that PA-X possibly reduced NS1 expression, thereby interfering with NS1’s shutoff activity. PA-X was reported to specifically target host RNA pol II transcripts [19]. However, the effect of PA-X on expression of viral mRNAs has not been determined. Therefore, we further analyzed the RNA-Seq data for the expression of viral mRNAs (Fig 8A). Our data indicate that PA-X and NS1 mutant viruses had differential effects on expression of each viral mRNA segment. Further analyses on the effect of shutoff-active NS1 (Fig 8B) and PA-X (Fig 8C) on expression of each viral mRNA segment were demonstrated. Intriguingly, the Cal [NS1_high-PAX_high] virus, which expressed normal amount of PA-X with shutoff-active NS1, demonstrated drastically low expression of NS1 and NEP mRNAs compared to other viruses (Fig 8B and 8C). This reduction of NS mRNAs were not observed in cells infected with Cal [NS1_low-PAX_high], Cal [NS1_low-PAX_low] or Cal [NS1_high-PAX_low] viruses, indicating that both shutoff-active NS1 and PA-X are required for an extensive reduction of NS mRNA expression.

**Fig 9:**
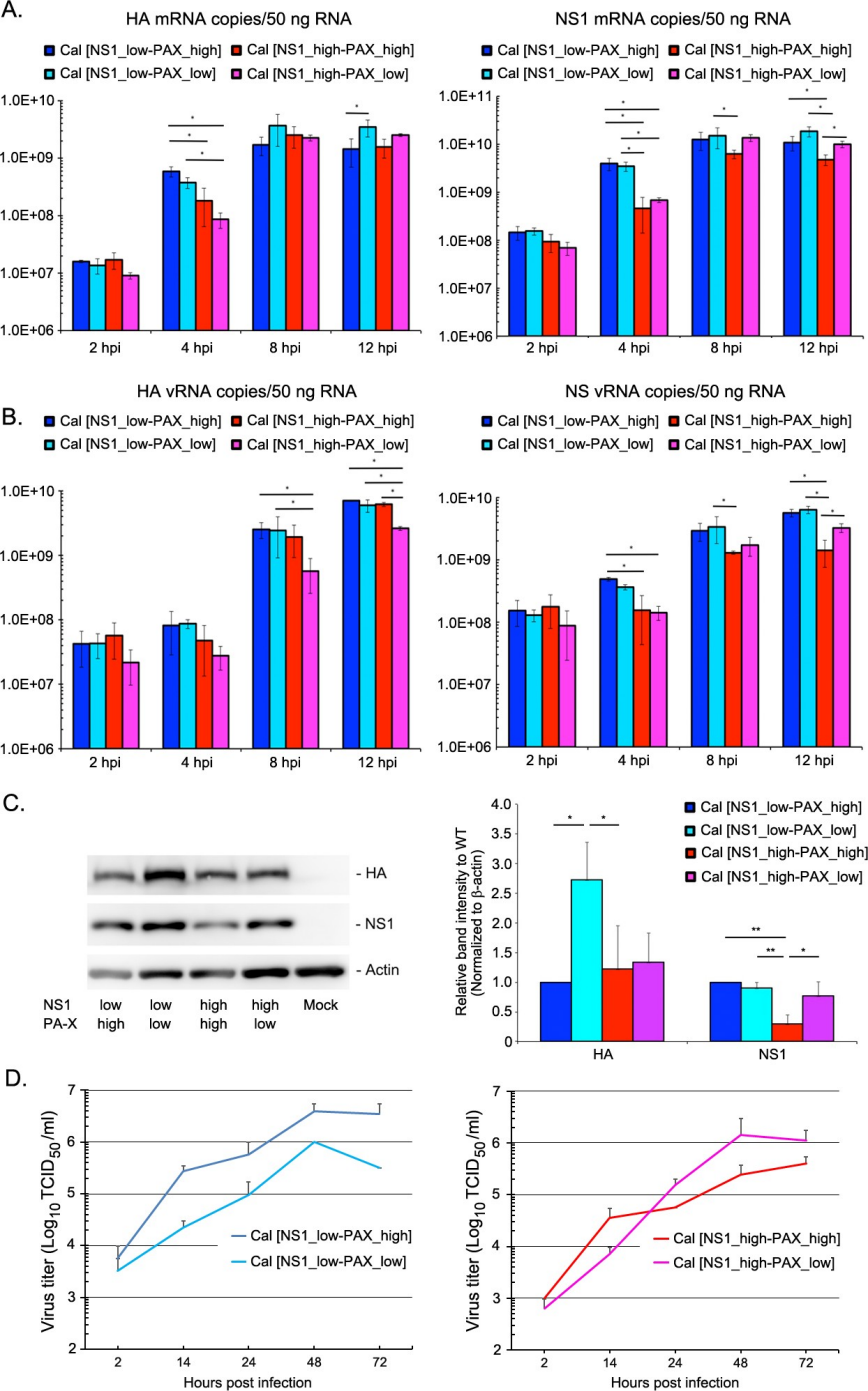
Expression of mRNA, vRNA and proteins of NS1 and HA, and growth of the viruses in Calu-3 cells. A549 cells were infected with the viruses at MOI of 2 and viral mRNA (A) and vRNA (B) were quantitated by qRT-PCR at various times after infection. The data represent averages with standard deviations (n = 3). *, P<0.05. (C) HA and NS1 protein expression in infected A549 cells were determined by Western blot analysis. Cellular actin protein was detected as a loading control. Left figure is a representative of three independent experiments and bar graph on right shows relative expression level (n = 3). *, P<0.05, **, P<0.01. (D) Multi-step growth of the viruses were determined in human airway Calu-3 cells. Cells were infected at MOI of 0.1 and released progeny virions in the culture supernatants at various time points were titrated. The data represent averages with standard deviations (n = 3).

We verified the gene expression of NS1 and HA mRNAs in infected A549 cells by qRT-PCR at various times after infection (Fig 9A). Comparison of the effect of PA-X on NS1 and HA mRNA levels (Cal [NS1_low-PAX_low] vs. Cal [NS1_low-PAX_high] and Cal [NS1_high-PAX_low] vs. Cal [NS1_high-PAX_high] viruses) indicates that PA-X also reduced the expression of viral mRNAs especially at later time points when more PA-X are accumulated in the cells. Consistent with the RNA-Seq data, the lowest NS1 mRNA levels were detected in Cal [NS1_high-PAX_high]-infected cells by qRT-PCR. Therefore, in the presence of shutoff-active NS1, PA-X likely contributed to a reduction of NS1 mRNA. To determine if this reduced NS1 mRNA is common to NS gene, we also determined the level of NS vRNAs in infected cells (Fig 9B). Interestingly, NS vRNA levels were also the lowest in Cal [NS1_high-PAX_high]-infected cells, suggesting the possibilities that PA-X also reduced replication of NS RNAs or targeted NS RNAs in the presence of shutoff-active NS1. To investigate the effect of NS1 and PA-X on viral gene expression in different host species, viral vRNA and mRNA levels were also measured in chicken DF-1 cells and demonstrated a similar trend (S3 Fig). To further verify the reduction of NS1 expression in Cal [NS1_high-PAX_high] virus infected cells, we quantified the NS1 and HA protein levels in infected human A549 cells (Fig 9C). As expected from qRT-PCR data, expression of NS1 was significantly reduced only in cells infected with Cal [NS1_high-PAX_high]. To determine the effect of NS1 and PA-X expression on viral growth in human airway cells, we infected Calu-3 cells with the viruses at an MOI of 0.1 and measured the virus growth at various time points. As we previously reported, reduced expression of PA-X attenuated growth and spread of wild-type Cal [NS1_low-PAX_high] virus (Fig 9D left) [14]. However, there was a trend towards enhanced growth and spread by reduced PA-X expression in the presence of shutoff active NS1, although statistical analysis showed no significance (Fig 9D right). These results suggest that although PA-X enhances growth and spread of the virus lacking the NS1 shutoff activity, it negatively impact on the growth of viruses expressing shutoff active NS1, possibly due to the restricted expression of NS1 that specifically target and regulate host innate responses (Fig 5A). Collectively, our findings indicate that NS1 shutoff activity plays a critical role in blocking antiviral responses in human hosts. However, the shutoff activity of NS1 can be less efficient due to suppression of NS mRNAs by PA-X shutoff activity. Overall, our data suggest that influenza viruses need to adjust their NS1 and PA-X activities to achieve maximum effect on counteracting host responses.

## Discussion

Influenza A viruses express two accessory proteins, NS1 and PA-X, to induce general host shutoff, which work through different mechanisms [7, 11]. Interaction of NS1 with CPSF30 prevents processing of pre-mRNA in the cell nucleus, while PA-X directly degrades RNA Pol II transcripts in both nucleus and cytoplasm of an infected cell [7, 8, 12, 19, 35, 36]. Both NS1 and PA-X contribute to antagonizing host antiviral and immune responses [4, 11, 14, 15, 37, 38]. However, their shutoff activities vary between virus isolates. Most seasonal human H1N1 and H3N2 viruses express shutoff-active NS1, but their PA-X shutoff activities are relatively low compared to avian-origin viruses due to various mutations in the N-terminal domain of PA gene [9, 18]. The 2009 pH1N1 strains express shutoff-inactive NS1 due to amino acid substitution in CPSF30 binding residues, while their PA-X shutoff activities are highly active due to their avian-origin PA gene [9, 12, 18, 39]. Influenza viruses rely on cellular machinery for genome replication and viral protein synthesis; therefore, the viruses likely need to adjust their shutoff activities to create an optimum cellular environment for efficient viral growth. In this study, we investigated the functional interplay between influenza PA-X and NS1 proteins on viral and host gene expression in infected human and avian cells and identified preferential cellular mRNA targets and the biological pathways associated with PA-X and NS1 shutoff activities. We found that i) PA-X strongly inhibits host protein synthesis in human and avian cultured cells, while the effect of active NS1 on general host protein synthesis was predominantly observed in avian cells (Fig 2); ii) PA-X preferentially targets cellular mRNAs involved in protein modification, metabolism and repair processes, while shutoff-active NS1 specifically targets genes involved in antiviral responses and strongly suppresses induction of IFN, interleukin, chemokine and class I MHC genes (Figs 4 and 5, S1 and S2 Tables); iii) shutoff-active NS1 together with PA-X predominantly repressed viral NS1 and NEP mRNA expression (Figs 8, 9 and S3); and iv) the virus expressing shutoff-active NS1 with a limited amount of PA-X (Cal [NS1_high-PAX_low]) whose shutoff activity mimics that of seasonal human viruses, is the most potent in antagonizing antiviral and innate immune responses in human cells (Figs 5 and 6).

Our data evidently indicate that PA-X has a strong impact on host gene expression (Fig 2). Specifically, infection with influenza viruses possessing highly active PA-X shutoff activity, regardless of shutoff activity from NS1, results in a rapid decline in host protein synthesis in A549 cells (Fig 2). This result is consistent with the observation that PA-X expressed from cDNA suppressed reporter gene expression more efficiently than shutoff-active NS1 [12]. Expression of PA-X is highly conserved among influenza A viruses [17] and it induces host shutoff in broad species [11, 14, 15, 40, 41]. In contrast, the CPSF30 binding residues of NS1, which are crucial for inducing host shutoff, are less conserved among animal isolates [9, 42]. The biological and evolutionary reasons why the influenza viruses maintain PA-X expression and its shutoff activity while having differential NS1 shutoff activities across the strains are not actually known. Expression of highly active PA-X and shutoff-active NS1 exhibits the strongest host shutoff activity in avian DF-1 cells; however, we speculated that this condition might not be optimum for avian or animal virus growth within their hosts due to their dependence on host machinery.

Both NS1 and PA-X were reported to induce general shutoff, still whether their shutoff activities are specific to certain host genes is not known. Our RNA-Seq and pathway enrichment analysis indicated that shutoff-active NS1 specifically targets the host mRNAs related to IFN and cytokine signaling pathways (Figs 3A and 4, S1 and S2 Tables). The GO analysis of NS1 showed strikingly high combined scores to genes involved in type I IFN signaling pathway. These genes were significantly reduced in cells infected with Cal [NS1_high-PAX_low] (Fig 5A). Importantly, this specific targeting cannot be solely explained by its ability to block pre-mRNA processing through CPSF30 binding, since this general shutoff mechanism should apply to global cellular mRNAs. Our data suggest that the NS1-CPSF30 complex either has an additional role in blocking innate signaling pathways or inhibits the processing of specific mRNAs involved in innate signaling. NS1 is known to interact with TRIM25, which ubiquitinates and activates RIG-1, subsequently inhibiting innate signaling pathway. We determined the TRIM25 interaction with wild-type and mutant NS1, and detected no difference in their binding. Also, wild-type and all the mutant viruses we tested suppressed IRF3 phosphorylation and its nuclear translocation in infected cells, suggesting that the NS1 mutation that allows for CPSF30 interaction has little effect on innate signaling that mediates IRF3 activation and translocation (Fig 7). A recent structural analysis showed that NS1 interferes with the correct positioning of the TRIM25 PRYSPRY domain required for RIG-I ubiquitination [32]. Cal NS1 contains E96/E97 residues in the effector domain (ED), which have been shown to be important for the complex formation with TRIM25 [31]. Therefore, our data suggest that NS1-CPSF30 complex might have an inhibitory effect during the transcription of innate genes in the nucleus.

The NS1-CPSF30 complex seems to be important for efficient viral growth and spread in mammalian host. A previous report indicated that NS1 mutations at 103 and 106 in H5N1 virus significantly enhance systemic growth and virulence in mice [42]. In the crystal structure, two NS1 ED and two CPSF30-F2F3 fragments formed a complex [8]. One NS1 ED interacts with one CPSF30-F2F3 through residues 108, 125 and 189, and with other molecules through residues 103 and 106, which seems to stabilize the complex formation. This tetramer complex formation likely promotes effective inhibition of antiviral responses, but could also have negative impact on viral replication as discussed below. Interestingly, sequence data available in the Influenza Research Database indicate that although early pH1N1 isolates contain 108R, 125E and 189G, pH1N1 viruses mutated at residue 125 from E to D emerged in 2015 and became dominant thereafter (S4 Fig). At this stage, it is unclear if this single mutation alters the interaction with CPSF30 or its shutoff activity. However, pH1N1 may continue to mutate in its NS1 and PA-X residues during seasonal infections to enhance its activity to specifically inhibit innate and cytokine responses in human hosts.

In contrast to NS1 which specifically targeted innate genes, PA-X preferentially targeted genes involved in protein modification or catabolic processes, which may have additional impact on its ability to suppress host protein expression in infected cells (Fig 2). However, the combined scores of these pathways are relatively low compared to what the biological pathways detected in NS1-shutoff active viruses (S1 and S4 Tables). These statistical values indicate how likely the target genes in a group are associated with a particular pathway. Hence, it is also possible that PA-X has more general targets than shutoff-active NS1 and is not truly associated with any specific pathway.

Viral shutoff could also negatively affect viral gene expression because influenza viruses totally rely on cellular resources for their replication cycle. In fact, we observed reduced expression of viral mRNAs on the virus expressing highly active PA-X and shutoff-active NS1 (Cal [NS1_high-PAX_high], Figs 8 and 9). This suppression of viral mRNAs was detected both in human A549 and chicken DF-1 cells (Figs 8, 9 and S3). Interestingly, NS genes (NS1 and NEP mRNAs) were the most affected in the virus expressing active PA-X and shutoff-active NS1, indicating both proteins act together to reduce NS mRNA expression (Fig 8B and 8C). This reduction was common between NS1 and NEP mRNAs, suggesting that these NS mRNAs include *cis*-acting sequence(s) that can be a target of PA-X in a presence of shutoff-active NS1. Previous reports suggest a direct interaction of NS1 protein with NS mRNAs [43, 44], which may allow PA-X targeting NS mRNAs through a direct or indirect interaction with shutoff-active NS1. In conclusion, our data support the idea of a functional interplay between PA-X and NS1 proteins in counter-regulating expression of viral genes.

## Materials and methods

### Cells and viruses

Madin-Darby canine kidney (MDCK), human lung adenocarcinoma epithelial A549 and Calu-3, chicken fibroblast DF-1 cells were obtained from American Type Culture Collection and maintained in Dulbecco’s modified Eagle’s medium with L-glutamine, 4.5 g/L glucose and sodium pyruvate (DMEM; Corning) supplemented with 8% fetal bovine serum (FBS, Seradigm) and HEPES (Gibco). A/California/04/2009 wild-type (Cal [NS1_low-PAX_high]) and Cal [NS1_low-PAX_low] viruses were previously generated by reverse genetics [14]. Sendai virus Cantel strain was propagated in 10-day-old embryonated chicken eggs.

### Plasmids

pPolI-CalNS3m was created by subcloning HindIII/pPuMI fragment of pCAGGS-CalNS3m [12] to pPolI-CalNS1m (R108K), which was generated by site-direct mutagenesis. pPolI-CalPA-XFS was previously constructed by site-directed mutagenesis [14]. Other pPolI and pCAGGS vectors for virus rescue were described previously [14, 18, 45].

### Virus rescue

Cal [NS1_high-PAX_high], and Cal [NS1_high-PAX_low] viruses were rescued by a 12-plasmid rescue system [46]. Rescued viruses were plaque purified in MDCK cells, and stock virus was propagated in 10-day-old embryonated chicken eggs. Mutations in PA and NS sequences were confirmed by sequence analysis. The viruses were titrated in MDCK cells, A549, Calu-3 and DF-1 cells by immunofluorescence analysis of the NP [47].

### Viral growth kinetics

Multi-step growth kinetics of the viruses was analyzed in MDCK and Calu-3 cells. In brief, confluent cells were infected with the viruses at an MOI of 0.05 (MDCK) or 0.1 (Calu-3) for 1 h and cultured in DMEM containing 0.15% bovine serum albumin (BSA) with N-tosyl-L-phenylalanine chloromethyl ketone (TPCK)-treated trypsin (2 μg/ml) for MDCK cells or without trypsin for Calu-3 cells. At various h pi, 10% of the cultured supernatant was collected and titrated in MDCK cells. Viral titers were expressed as 50% tissue culture infective dose (TCID_50_)/ml. A single step growth of the viruses was determined in A549 and DF-1 cells. Confluent cells were infected with the viruses at MOI of 2 for 1 h. After washing, infected cells were cultured in DMEM containing 0.15% BSA without trypsin. Culture supernatants were collected at 2 and 18 h pi, and virus titers were titrated in MDCK cells.

### qRT-PCR

To quantitate HA and NS vRNAs and mRNAs, total RNAs were extracted from infected cells using Illustra RNAspin Mini (GE Healthcare) or Trizol (Invitrogen). cDNAs complementary to the influenza vRNA and mRNA were synthesized using RevertAid First Strand cDNA Synthesis (Thermo Scientific) using 50 ng total RNA and specific primers. Primers for reverse transcription (RT) were as follows: (i) Cal HA vRNA (vRNAtag-HA1249F): GGCCGTCATGGTGGCGAATAGTTCACAGCAGTAGGTAAAGAGTTC; (ii) Cal HA mRNA (mRNAtag-HA1761R): CCAGATCGTTCGAGTCGTTTTTTTTTTTTT TTTCTCATGCTTCTGAAATCCTAATG; (iii) Cal NS vRNA (vRNAtag-NS541F): GGCCGTCATGGTGGCGAATAGGATGTCAAAAATGCAGTTG; (iv) Cal NS mRNA (mRNAtag-NS874R): CCAGATCGTTCGAGTCGTTTTTTTTTTTTTTTTTATCATTAAA TAAGCTG. Real-time qPCR was performed with SYBR Green (Thermo Fisher Scientific). qPCR cycle conditions were 95°C for 10 min, followed by 40 cycles of 95°C for 15 s and 60°C for 1 min [48]. Primers for qPCR were as follows: (i) Cal HA vRNA forward primer (vRNAtag): GGCCGTCATGGTGGCGAAT; (ii) Cal HA vRNA reverse primer (HA1357R): AGTTCGGCATTGTAAGTCCAAATGTCC; (iii) Cal HA mRNA forward primer (HA1652F): ATTGGTACTGGTAGTCTCCCTG; (iv) Cal HA mRNA reverse primer (mRNAtag): CCAGATCGTTCGAGTCGT; (v) Cal NS vRNA forward primer (vRNAtag): GGCCGTCATGGTGGCGAAT; (vi) Cal NS vRNA reverse primer (NS681R): TCTGCTCTGGAGGTAGTGAAGGTCTCC; (vii) Cal NS mRNA forward primer (NS775F): AGTTTCGAACAAATAACATTTATGC; (viii) Cal NS mRNA reverse primer (mRNAtag): CCAGATCGTTCGAGTCGT. DNA standards for HA or NS vRNA and mRNA were prepared from purified PCR products of the genes containing both vRNAtag and mRNAtag.

For qRT-PCR of IFN-λ1, IFN-β and IL6, total RNAs were extracted at 16 h pi. cDNAs were synthesized using RevertAid First Strand cDNA Synthesis (Thermo Scientific) using 1 μg total RNA and oligo-dT primer. Real-time qPCR was performed with SYBR Green as above. Primers for qPCR were as follows: (i) Human IL-6 forward primer (human-IL6-rt289F): GGTACATCCTCGACGGCATCT; (ii) Human IL-6 reverse primer (human-IL6-rt369R): GTGCCTCTTTGCTGCTTTCAC; (iii) Human IFN-λ1 forward primer (humanIFNL1-148F): TTCAAGAAGGCCAGGGACG; (iv) Human IFN-λ1 reverse primer (humanIFNL1-245R): AGAAGCCTCAGGTCCCAATT; (v) Human IFN-β forward primer (humanIFNb-RT617F): TTACAGGTTACCTCCGAA ACTGAA; (vi) Human IFN-β reverse primer (humanIFNb-RT695R): GGTTGAAGA ATGCTTGAAGCAA.

DNA standards for cellular mRNA were prepared from purified PCR products of target genes. RT reaction was performed using total RNAs extracted from A549 cells infected with wild-type Cal [NS1_low-PAX_high]) and oligodT primer. Primers used for the PCR are (humanIL6-1F): ATGAACTCCTTCTCCACAAGCG, and (humanIL6-639R): CTACATTTGCCGAAGAG CCCTC, (humanIFNλ1-1F): ATGGCTGCAGCTTGGACCG, and (humanIFNλ1-603R): TC AGGTGGACTCAGGGTGG, (humanIFNβ-76F-NotI): ACTCGAGCGGCCGCCAATGAC CAACAAGTGTCTCCTCCAAA, and (humanIFNβ-753R-HindIII): AAACTTAAGCTTTT CTAGTGTCCTTTCATATGCAGTA.

### Enzyme-linked immunosorbent assay (ELISA)

Secreted cytokines released from infected cells were quantitated using human IL-6 uncoated ELISA kit (Thermo Fisher Scientific; 88–7066) and human IL-29 (IFN lambda 1) uncoated ELISA kit (Thermo Fisher Scientific; 88–7296). We followed the manufacturers’ procedures and used 100 ul of culture supernatant for detection.

### Metabolic labeling

Confluent human A549 and chicken DF-1 cells in a 24-well plate were either left uninfected or infected with the viruses at an MOI of 2 (A549) or MOI of 1 (DF-1) for 1 h and incubated in DMEM containing 0.15% BSA. At various times after infection, cells were washed and cultured in methionine, cysteine-free DMEM media (Gibco) for 30 min. Then, cells were labeled with ^35^S-Met/Cys (25 μCi, PerkinElmer) in the same medium for 30 min at 37°C. Cells were lysed with 50 ul of NP-40 lysis buffer (25 mM Tris-HCl [pH 7.4], 150 mM NaCl, 1 mM EDTA, 1% NP-40, and 5% glycerol), and 15 ul of the radiolabeled lysates were resolved in 10% sodium dodecyl sulfate (SDS)-polyacrylamide gels. The dried gels were exposed on a phosphor screen and visualized using Personal Molecular Imager (Bio-Rad). Volume analysis was performed using Quantity One 1-D analysis software (Bio-Rad).

### Co-immunoprecipitation and western blot analysis

A549 cells infected with the viruses at MOI of 5 in 6-well plate were cultured for 16 h at 37 ^o^C. Cells were lysed with 300 ul of IP lysis buffer (Pierce) and reacted with 20 ul of Dynabeads (Invitrogen) reacted with 2 ul of anti-NS1 Ab (NR44426, BEI resources). Bound protein were eluted with SDS-PAGE sample buffer and analyzed by Western blotting. Immunoprecipitated proteins or cell lysates were applied to a 12% SDS-PAGE gel and transferred onto a polyvinylidene difluoride (PVDF) membrane (Millipore). The blot was blocked with 2% dry milk in Tris-buffered saline with Tween 20 (TBST) and then incubated with primary antibodies as follows: rabbit anti-TRIM25 monoclonal antibody (mAb) (1:1,000, abcam), rabbit anti-NS1 polyclonal antibody (1:500, Invitrogen), and mouse anti-β-actin mAb (1:5,000; Cell Signaling). After washing, cells were incubated with horseradish peroxidase (HRP)-conjugated goat anti-mouse IgG (1:10,000; Santa Cruz) or anti-rabbit IgG (1:5,000; Thermo Fisher). Target proteins were visualized using SuperSignal West Femto maximum sensitivity substrate (Thermo Scientific). Images were captured using the ChemDoc XRS system (Bio-Rad) and analyzed using Quantity One 1-D analysis software (Bio-Rad). For the analysis of IRF3 activation, A549 cells in 6-well plate were infected with the viruses at MOI of 5 and cultured for 6 h at 37 ^o^C. Cells were lysed with the IP lysis buffer and analyzed for IRF3 activation using mouse anti-IRF3 (1:500; eBioscience), rabbit anti-IRF3 (phospho S386)(1:500; abcam), mouse anti-SeV NP cocktail (1:1,000; [49]), and mouse anti-M1 mAb (1:1,000; Abcam). For the analysis of viral protein level, mock- and virus-infected cells in 12-well plate were lysed with 100 ul of cell lysis buffer (20 mM HEPES [pH 7.5], 1.5 mM MgCl2, 500 mM NaCl, 0.2 mM EDTA, 1% Triton X-100, and 20% glycerol). Fifteen ul of the cleared lysates were analyzed by Western blotting using a cocktail of mouse anti-influenza A HA mAb NR42019, NR42020, NR28666, NR28667, and NR28668 (1:1,000; BEI Resources), and mouse anti-influenza A NS1 [10C7] mAb (1:1,000; Kerafast).

### Immunofluorescence assay

A549 cells infected with the viruses at MOI of 5 for 6 h were fixed with 3.5% paraformaldehyde in PBS, and permeabilized with methahol/acetone (1:1) for 10 min. Cells were reacted with mouse anti-IRF3 mAb (1:1,000, eBioscience) followed by anti-mouse IgG conjugated with AlexaFluor 594 (1:1,000, Invitrogen). Cells were counterstained with DAPI. Images were obtained using an Olympus IX50 inverted fluorescence microscope with a 60x oil immersion objective.

### RNA sequencing (RNA-Seq)

Confluent A549 cells in a 12-well plate were either left uninfected or infected with the viruses at an MOI of 2 for 16 h in triplicates. Total RNAs were extracted using Illustra RNAspin Mini (GE Healthcare). RNA concentration and quality were measured using an Agilent 2100 Bioanalyzer, showing that RNA Integrity Number (RIN) values of > 7.0, OD260/280 = 2.0–2.2 and 28S: 18S rRNA > 1.0. mRNA was purified from 200 ng total RNA with oligo-dT magnetic beads and fragmented. First-strand cDNA synthesis was performed with random hexamer priming followed by second-strand cDNA synthesis. End repair and 3’ adenylation was then performed on the double-stranded cDNA. Illumina adaptors were ligated to both ends of the cDNA, purified by gel electrophoresis, and amplified with PCR primers specific for the adaptor sequences to generate amplicons of approximately 200–500 bp in size. The amplified libraries were hybridized to the Illumina single-end flow cell and amplified using the cBot (Illumina) at a concentration of 8 pM per lane. Single-end reads of 100 nt were generated for each sample and aligned to the organism-specific reference genome. RNA-sequencing was performed using the Illumina HiSeq2500 sequencer.

Sequence reads were cleaned and adapter trimmed using trimmomatic-0.36. The reads quality were checked by FastQC before mapping them to the human reference genome (GRCh38.p7, primary assembly + gencode 25 annotation) or the influenza reference genome (Influenza A/ California/04/2009 (H1N1); Accession: PRJNA363070) with STAR2.5.2b. Raw read counts were obtained using featurecounts from the subread 1.5.0p3 package and gencode 25 human gene annotations using only uniquely aligned reads. DESeq2-1.16.1 was used to perform differentially expressed genes (DEG) analysis. DESeq2 performs an internal normalization where read counts for every gene in each sample were divided by geometric mean. The median of the normalization ratio is the size factor for that sample. This procedure corrects for library size and RNA composition bias. Differentially expressed genes were further defined as those genes that have absolute fold change value >2.0 with an adjusted P-value threshold of 0.05.

Functional enrichment analysis of DEG was performed with Enrichr analysis tool (http://amp.pharm.mssm.edu/Enrichr/). To associate a set of differentially expressed genes with a functional biological term, Gene Ontology terms were assigned using GO biological processes 2017b annotation set. GO terms were ranked by a combined score (ln(P-value) × Z-score); the P-value computed using the Fisher exact test.

### Statistical analysis

Statistical analysis for all experiments, excluding the RNA-Seq study, was performed using one-way ANOVA followed by Tukey’s multiple comparison test (JMP Pro 12). A P-value of < 0.05 was considered statistically significant.

### Ethics statement

Embryonated chicken eggs were obtained from Charles River. Ten days-old embryos were inoculated with the viruses and cultured for three days before harvesting the allantoic fluids.

## Supporting information

S1 Fig Processing statistics of transcriptomic data (RNA sequencing) and validation of RNA samples. (A) Processing statistics for transcriptomic analysis of the 15 RNA samples including three biological replicates of uninfected condition (mock), and Cal [NS1_low-PAX_high], Cal [NS1_low-PAX_low], Cal [NS1_high-PAX_high], and Cal [NS1_high-PAX_low] infected A549 cells at MOI of 2 for 16 h. The processing statistics include number of raw, cleaned, removed, uniquely mapped, non-uniquely mapped and, no feather count of sequence reads of each sample. (B) Sample-to-sample distance matrix of RNA-Seq analysis. A heatmap shows the hierarchically clustered Euclidean distances between samples from the regularized log transformation of the normalized count data. The scale on the right demonstrates the arbitrary unit of distance between samples in which the dark color represents less distance (more similarity) and the light color represents greater distance between samples (less similarity). (C) Principal component analysis (PCA) plot representing the variance in the gene dataset. The samples shown in the 2D plane are spanned by their first two principal components of all samples including three replicates of mock, Cal [NS1_low-PAX_high], Cal [NS1_low-PAX_low], Cal [NS1_high-PAX_high], and Cal [NS1_high-PAX_low] infected conditions.
(TIF)

S2 Fig Differential expression of genes of individual virus-infected conditions with mock. (A) Volcano plots of differential gene expression between individual virus-infected conditions versus mock demonstrate the significance (-log_10_ P-value) on the y-axis and the magnitude of difference (log_2_ fold-change) on the x-axis. Genes with multiple test corrected P-value < 0.05 were colored according to the direction of the fold-change (green: down-regulation or red: up-regulation). A line was drawn at the unadjusted P-value of 0.05 for reference. (B) A table demonstrates the number of DEGs (adjusted P value < 0.05) and the number of at least two-fold up- or down-regulated DEGs of A549 cells infected with indicated viruses compared to mock. (C) Venn diagrams demonstrate genes that were at least two-fold up-regulated (left) or down-regulated (right) upon infection with Cal [NS1_low-PAX_high], Cal [NS1_low-PAX_low], Cal [NS1_high-PAX_high], and Cal [NS1_high-PAX_low] viruses compared to mock.
(TIF)

S3 Fig Quantities of HA and NS1 mRNAs and HA and NS vRNAs in infected DF-1 cells. Cells were infected with the viruses at MOI of 1 and viral mRNA (A) and vRNA (B) were quantitated by qRT-PCR at various times after infection. The data represent averages with standard deviations (n = 3). *, P < 0.05.
(TIF)

S4 Fig Sequence of pH1N1 NS1 residues at the CPSF30 interacting site. Sequence data of pH1N1 viruses isolated at various years were obtained from Influenza Research Database and the number of isolates having the indicated residues are shown.
(TIF)

S1 Table List of the DEGs included in each cluster of the suppressed genes by shutoff-active NS1. (XLSX)

S2 Table GO annotations related to genes suppressed by shutoff-active NS1. Enrichment analysis using GO Biological Process 2017b database of the 493 DEGs obtained from Fig 3A shows the pathways in which DEGs were suppressed by shutoff-active NS1 following virus infection. The ranking was based on the combined score, which was calculated from ln(P-value) × Z-score (Enrichr).
(XLSX)

S3 Table List of the DEGs due to PA-X expression. The level of gene expression in virus infected cells were compared with that of mock infected cells.
(XLSX)

S4 Table GO annotations related to genes suppressed by PA-X. Enrichment analysis using GO Biological Process 2017b database of the 156 DEGs obtained from Fig 3B shows the pathways in which DEGs were suppressed by PA-X following virus infection. The ranking was based on the combined score, which was calculated from ln(P-value) × Z-score (Enrichr).
(XLSX)
