# Supplementary figures and images for: Specificity and functional interplay between influenza virus PA-X and NS1 shutoff activity
Source: PLoS Pathog. 2018 Nov 29;14(11):e1007465. doi: 10.1371/journal.ppat.1007465 (PMC6289448; doi:10.1371/journal.ppat.1007465)

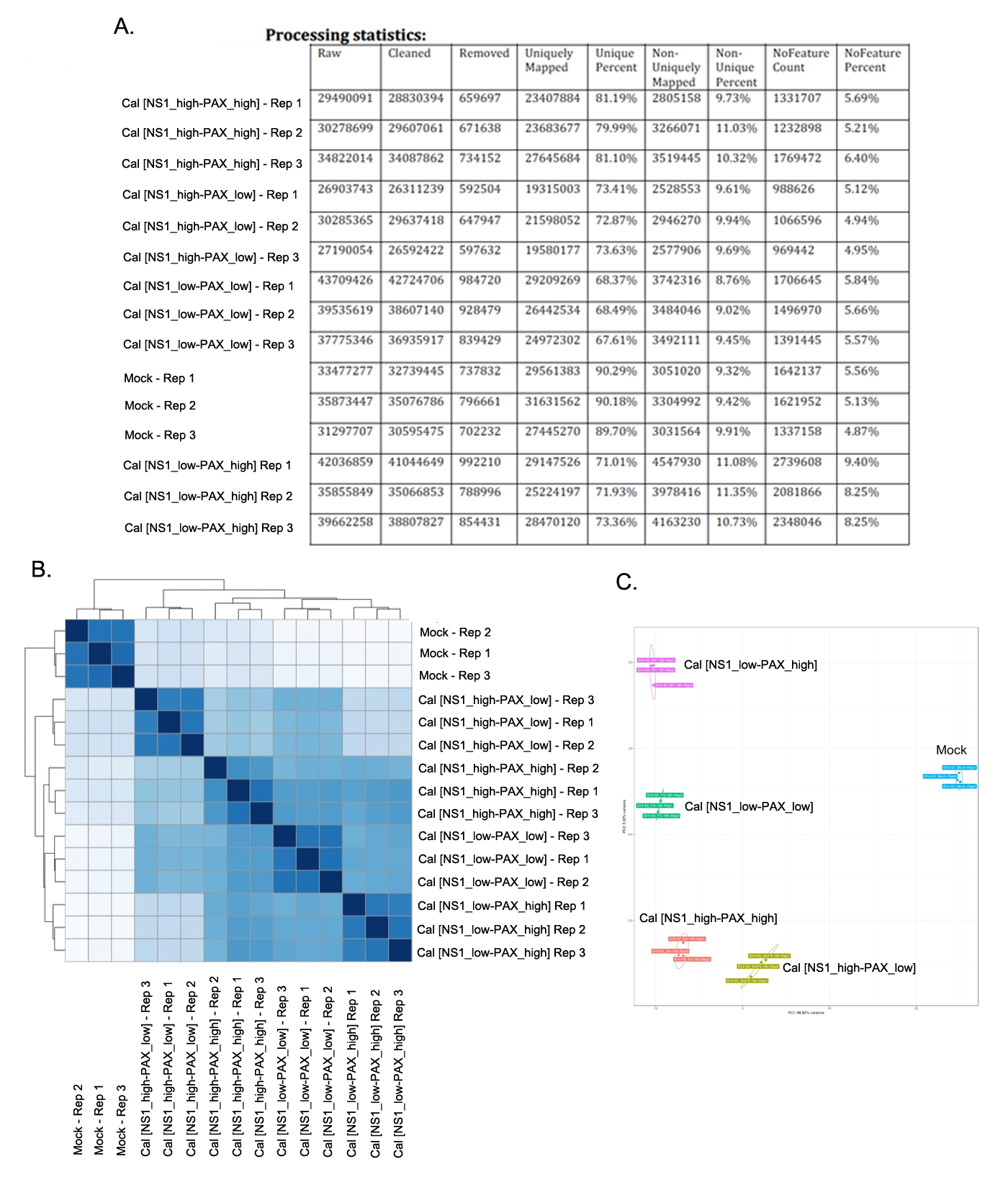

Supplement: S1 Fig — (A) Processing statistics for transcriptomic analysis of the 15 RNA samples including three biological replicates of uninfected condition (mock), and Cal [NS1_low-PAX_high], Cal [NS1_low-PAX_low], Cal [NS1_high-PAX_high], and Cal [NS1_high-PAX_low] infected A549 cells at MOI of 2 for 16 h. The processing statistics include number of raw, cleaned, removed, uniquely mapped, non-uniquely mapped and, no feather count of sequence reads of each sample. (B) Sample-to-sample distance matrix of RNA-Seq analysis. A heatmap shows the hierarchically clustered Euclidean distances between samples from the regularized log transformation of the normalized count data. The scale on the right demonstrates the arbitrary unit of distance between samples in which the dark color represents less distance (more similarity) and the light color represents greater distance between samples (less similarity). (C) Principal component analysis (PCA) plot representing the variance in the gene dataset. The samples shown in the 2D plane are spanned by their first two principal components of all samples including three replicates of mock, Cal [NS1_low-PAX_high], Cal [NS1_low-PAX_low], Cal [NS1_high-PAX_high], and Cal [NS1_high-PAX_low] infected conditions. (TIF) [file ppat.1007465.s001.tif]

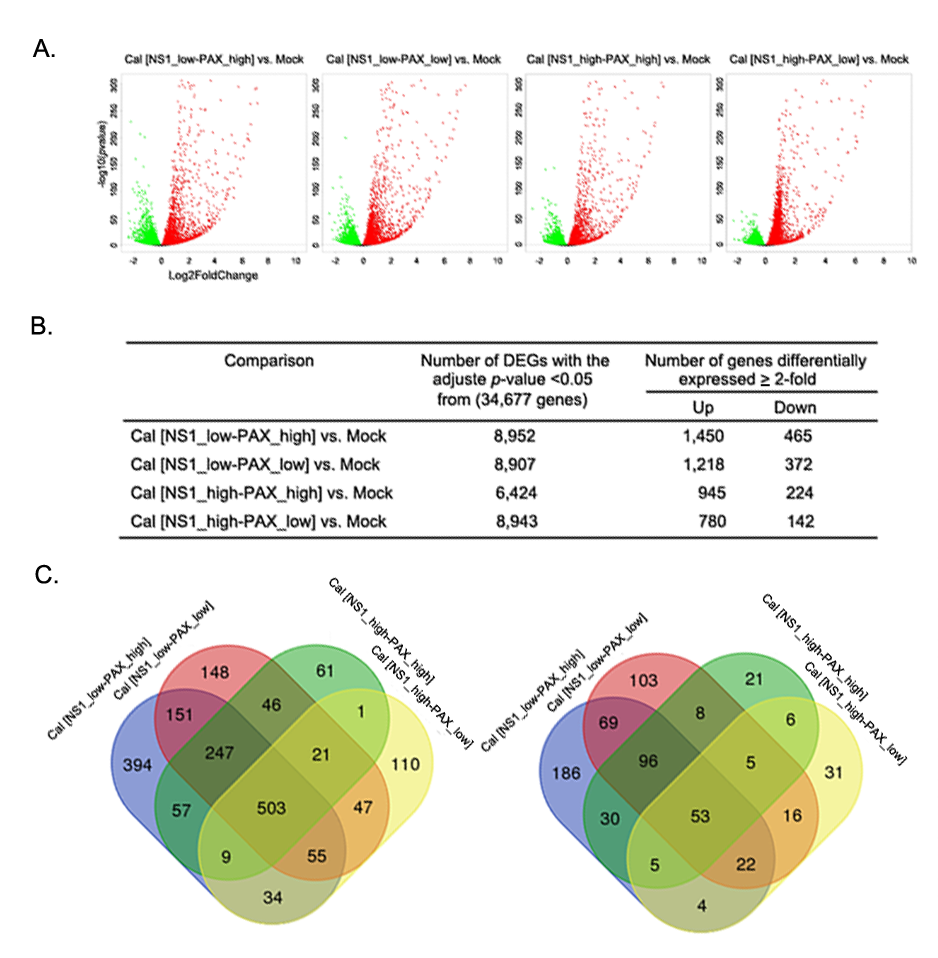

Supplement: S2 Fig — (A) Volcano plots of differential gene expression between individual virus-infected conditions versus mock demonstrate the significance (-log10 P-value) on the y-axis and the magnitude of difference (log2 fold-change) on the x-axis. Genes with multiple test corrected P-value < 0.05 were colored according to the direction of the fold-change (green: down-regulation or red: up-regulation). A line was drawn at the unadjusted P-value of 0.05 for reference. (B) A table demonstrates the number of DEGs (adjusted P value < 0.05) and the number of at least two-fold up- or down-regulated DEGs of A549 cells infected with indicated viruses compared to mock. (C) Venn diagrams demonstrate genes that were at least two-fold up-regulated (left) or down-regulated (right) upon infection with Cal [NS1_low-PAX_high], Cal [NS1_low-PAX_low], Cal [NS1_high-PAX_high], and Cal [NS1_high-PAX_low] viruses compared to mock. (TIF) [file ppat.1007465.s002.tif]

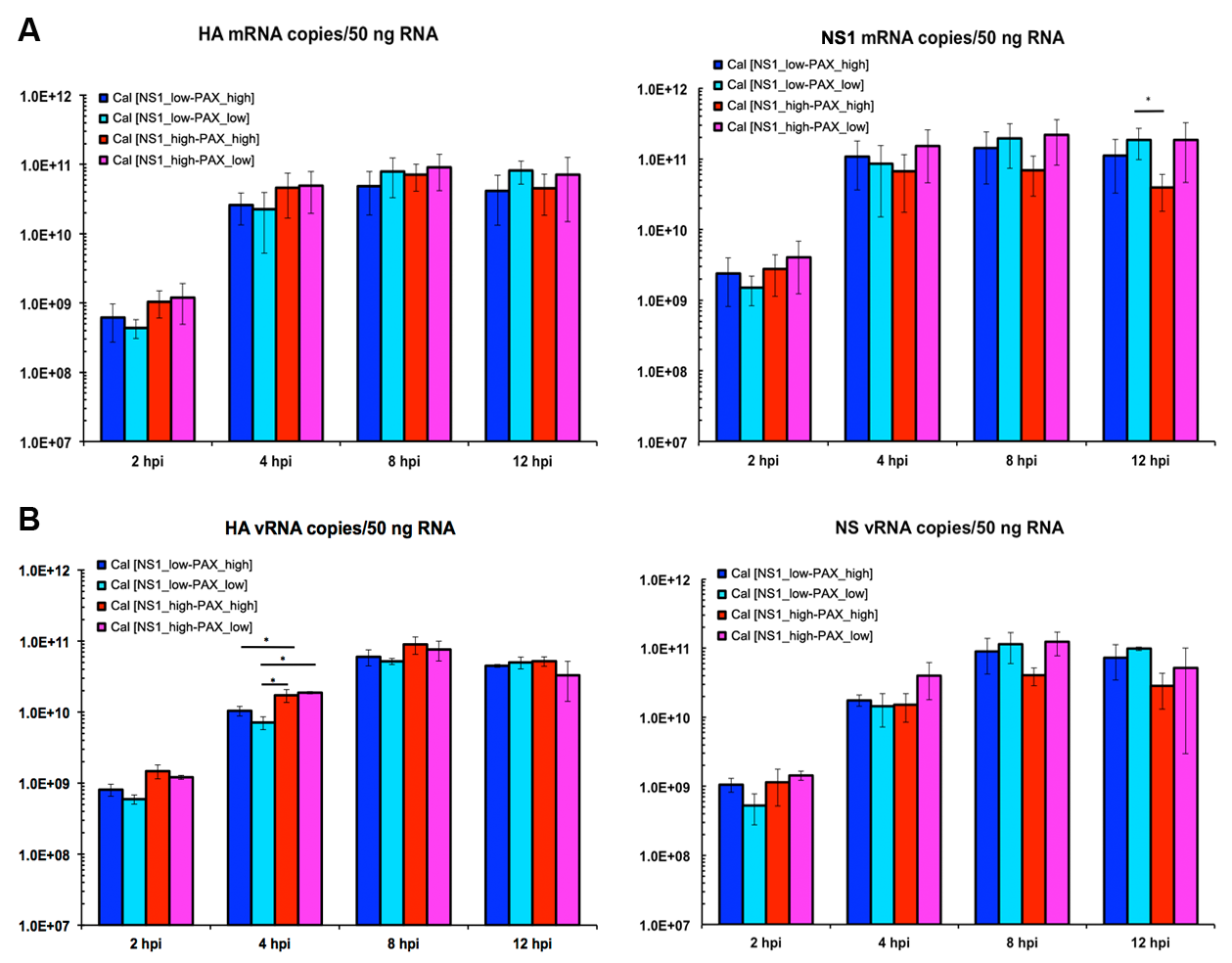

Supplement: S3 Fig — Cells were infected with the viruses at MOI of 1 and viral mRNA (A) and vRNA (B) were quantitated by qRT-PCR at various times after infection. The data represent averages with standard deviations (n = 3). *, P < 0.05. (TIF) [file ppat.1007465.s003.tif]

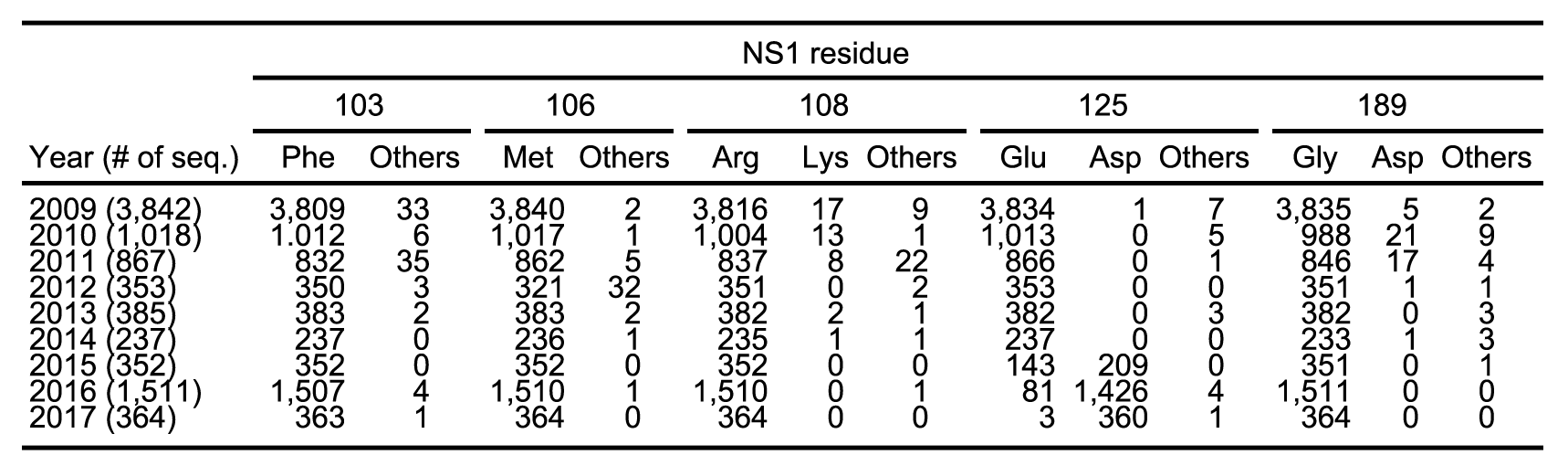

Supplement: S4 Fig — Sequence data of pH1N1 viruses isolated at various years were obtained from Influenza Research Database and the number of isolates having the indicated residues are shown. (TIF) [file ppat.1007465.s004.tif]
